# Pulsed Electrolysis Prevents Sulfur Poisoning for Sustained Sulfide Valorization

**DOI:** 10.1002/adma.73096

**Published:** 2026-04-17

**Authors:** Zhiyan Hou, Yangbo Ma, Yufeng Wu, Weijin Cao, Zhengxiao Guo, Changlong Wang

**Affiliations:** ^1^ State Key Laboratory of Materials Low‐Carbon Recycling College of Materials Science and Engineering Beijing University of Technology Beijing China; ^2^ Department of Chemistry The University of Hong Kong Hong Kong SAR China

**Keywords:** catalyst passivation mitigation, electrochemical sulfide oxidation, hydrogen and sulfur co‐production, pulsed electrolysis

## Abstract

Hydrogen sulfide (H_2_S), a toxic byproduct generated from metallurgy, incineration, and natural gas purification, poses serious environmental and health risks. Current treatments (e.g., the Claus process) are energy‐intensive and generate secondary waste. Electrochemical sulfide oxidation (SOR) offers an energy‐efficient alternative for simultaneous H_2_S removal and recovery of high‐purity hydrogen and sulfur, but its application is hindered by anode deactivation due to sulfur deposition. Here, we report a dynamic microenvironment engineering strategy using pulsed electrolysis (PE) to achieve sustainable SOR. Coupled with a Sc‐doped NiFe‐LDH electrocatalyst optimized for intermediate adsorptions, we achieve periodical modulation of metal‐sulfur redox, enabling efficient sulfur release and active site regeneration. This synergy enables continuous H_2_S destruction and hydrogen production for over 500 h with a Coulombic efficiency of 99.8% and a low energy consumption of 2.19 kWh m^−3^. Furthermore, using bio‐derived formic acid, the acidification process is capable of co‐production high‐purity sulfur (99.5%) and sodium formate. This integrated process, validated also with industrial syngas and seawater electrolyte, increases the overall profit by 121% to US$1,294.7 per tonne of hydrogen. Overall, this report demonstrates a circular and economically viable strategy for H_2_S treatment and resource recovery, which is also implacable to other electrochemical systems facing catalyst poisoning.

## Graphical abstract

Integrating pulsed electrolysis with Sc‐doped NiFe‐LDH enables sustainable sulfide valorization, achieving >500 h stability, 99.8% efficiency. Moreover, this technology produces high‐purity sulfur and sodium formate, boosting profit by 121% to $1,294.7 per tonne of hydrogen.

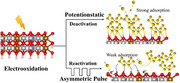

## 1 Introduction

Sulfur is widely used in optoelectronics and new energy fields (e.g., lithium‐sulfur batteries, sulfide solid‐state batteries), but the deconstruction process of electronic waste (phosphor powder, yttrium oxysulfide, zinc sulfide, sodium‐sulfur batteries, etc.) generates toxic hydrogen sulfide gas, which needs to be properly handled. Hydrogen sulfide (H_2_S) is a biologically toxic acidic gas, commonly found in critical industrial processes (e.g., natural gas purification, syngas production, refinery gas treatment, and mineral decomposition). The oil and natural gas extraction‐related industry is a major source of H_2_S generation, and its global output exceeding 85 million tons in 2024 [1, 2]. Moreover, in the high‐sulfur natural gas‐related scenario, the emission intensity (e.g., H_2_S concentration) even reaches up to 30%, posing serious challenges to environmental safety and energy utilization for its decomposition. Industrially, the Claus process and chelated iron method are employed for the reduction of the H_2_S emissions [3], addressing partially the problem (though the removal efficiency can be reached to 97%) but at the expense of a vast amount of water (and thus generating a substantial amount of sulfion‐rich wastewater) and other value‐less products. To further reduce the toxicity, alkaline H_2_S adsorption‐chemical precipitation cascade reaction is used [4]. However, this reaction often involves high reagent and energy consumption with relatively low conversion efficiency. This calls for the further development of new green technologies with both efficient desulfurization and high valuable‐resource recovery.

The electrochemical sulfion oxidation (SOR, S^2−^→S_0_ + 2e^−^), driven by renewable‐electricity at the anode, offers an efficient alternative for sulfion‐containing sewage treatment [5]. This technology generates valuable sulfur species under mild conditions and achieves high purification efficiency without the requirement of additional oxidants. On the other hand, SOR is thermodynamic favorable (0.142 V vs. RHE), and is also a welcomed substitution to oxygen evolution reaction (OER, 1.23 V vs. RHE) in electrochemical water splitting [6]. Thus, SOR enables both the anodic sulfide recovery (as elemental sulfur, S_0_) and cathodic green hydrogen (H_2_) production.

However, the problem associated with SOR is the strong metal‐sulfur bond over catalysts. On the one hand, the strong orbital interactions between sulfur species and the metal active sites facilitate the interfacial electron transfer. However, this high affinity of sulfur ions on catalyst surfaces also induces the corrosion and passivation effects [6, 7]. Moreover, the SOR processes with the chain‐growth mechanism, producing polysulfide intermediates with highly delocalized negative charges [8]. This charge delocalization increases the softness of the ions, potentially hindering the release of insulating sulfur species from the catalyst surface and aggravating passivation [9, 10]. As a result, most literature‐reported metallic electrodes exhibited poor stability at high current densities. A long‐standing challenge in SOR is the unresolved trade‐off between catalyst activity (requiring strong S_0_ adsorption) and stability (demanding rapid S_0_ desorption); this fundamentally limits the industrial adoption of SOR.

Recently, asymmetric pulse potential electrolysis, driven by non‐constant energy inputs (e.g., alternating high oxidation potentials and low open‐circuit intervals), enables the dynamic modulation of catalyst surface states and intermediate coverage through periodic interfacial perturbations [7, 11, 12]. We then hypothesize that pulsed electrocatalysis (PE) could dynamically resolve the conflict of SOR—that is, high‐potential pulses drive S^2−^ oxidation, while open‐circuit intervals facilitate S_0_ desorption via double‐layer discharge—to enhance both reaction efficiency and the catalyst's performance. Moreover, the anodic polysulfide generated ions (S_n_^2−^, 1< n < 8) at basified condition (pH 13–14) require substantial amount of acid (e.g., H_2_SO_4_ or HCl) for acidification, yielding elemental sulfur and additional waste salts [7, 12]; the process also needs to be modified for improved profit, enhanced sustainability and practical implementation.

Here, we report for the first time that the PE for SOR shows superior removal efficiency and long‐term stability using Sc‐NiFe‐LDH as the electrocatalyst. Compared to conventional constant‐potential electrocatalysis (CE), the alternating application of high oxidation potential and open‐circuit potential (OCP) in PE mode effectively accelerated the desorption of products and poisoning intermediates from the catalyst surface, thereby exposing fresh active sites for sustained electrolysis. At the PE mode, we achieved a high coulombic efficiency of 99.8%, a removal rate of 0.032 mmol min, and excellent stability (>500 h) for SOR. This SOR system operating at PE mode showed an energy consumption of 2.19 kWh m^−3^ H_2_, achieving a 57.7% reduction in energy consumption compared to traditional water splitting systems (5.18 kWh m^−3^).

Moreover, our system can also be scaled up, showcasing in H_2_S removal from simulated 2%H_2_S/Syngas with superior activity and stability. Furthermore, we also substitute the typical used mineral acids (e.g., H_2_SO_4_) with bio‐derived formic acid with the aim to further enable the circular conversion of waste sulfides into value‐added sulfur and sodium formate. In this way, techno‐economic analysis shows a 121% profit increase, rising from $585.90 to $1,294.71 per tonne of hydrogen. Thus, by dynamically regenerating active sites and preventing passivation, this potential‐pulsing strategy enhances reaction kinetics, and can be potentially extended to other electrocatalytic processes (e.g., oxygen evolution, alcohol oxidation, or biomass conversion). Our work demonstrates how potential‐driven dynamic control can shift reaction pathways and enable self‐regenerative, high‐performance catalytic systems.

## 2 Results and Discussions

### 2.1 Techno‐Economic Analysis Guides the Development of Sulfide Oxidation Reaction

**FIGURE 1:**
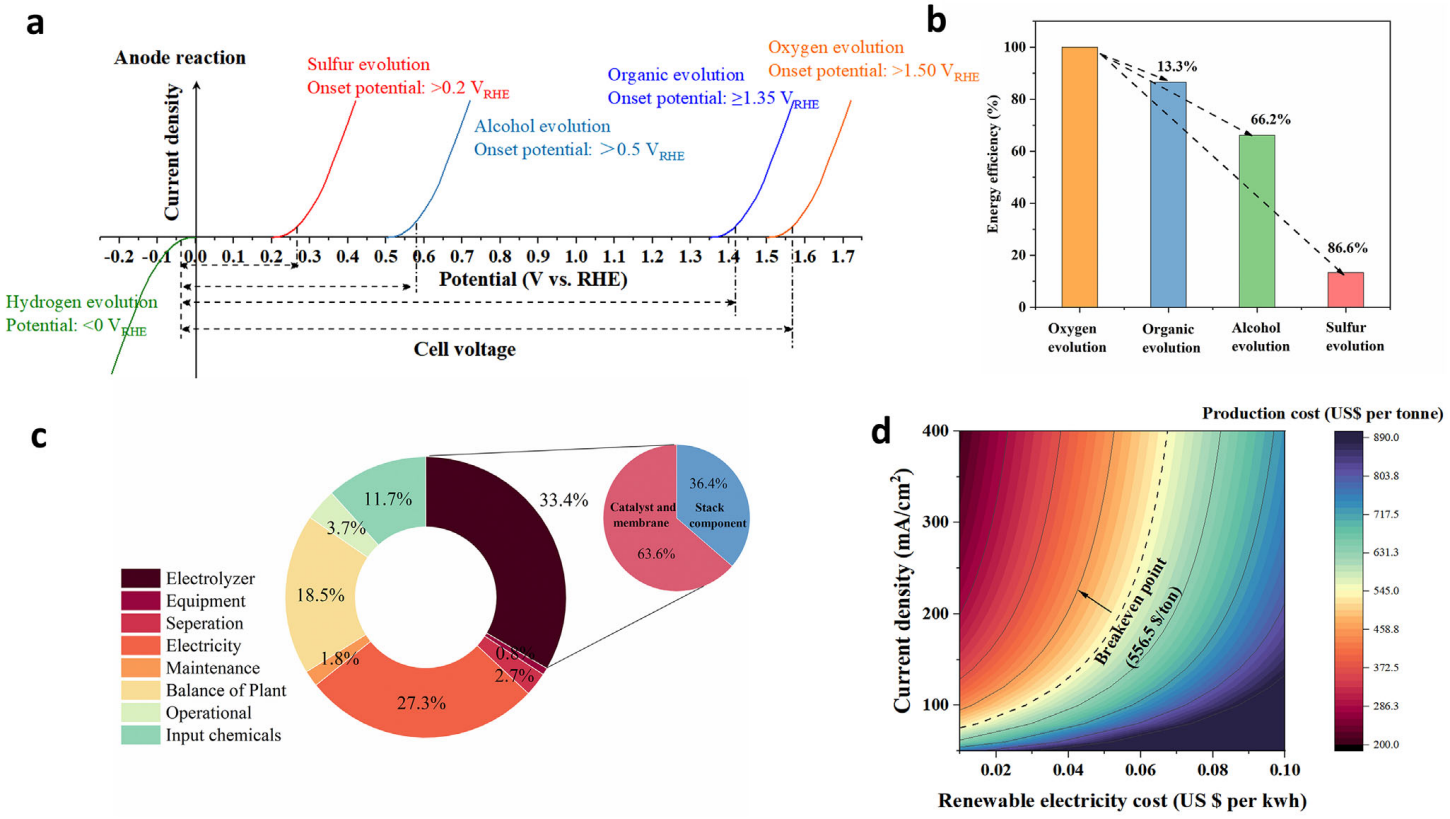
(a) Comparison of various anodic reactions with water electrolysis; (b) Reduction rate in energy efficiency compared to OER; (c) Cost distributions of the entire process for SOR at the given electricity price of US$0.03 kWh^−1^; (d) Levelized cost of co‐productions hydrogen and sulfur via SOR, as a function of current density and electricity cost.

Electrochemical oxidation offers mild reaction conditions with oxidative potentials and electrolytes finely tuned to oxidize the substrates to desired products. This includes the selective oxidation of alcohols (e.g., methanol and ethanol) [13, 14], biomass platform chemicals (e.g., 5‑hydroxymethylfurfural (HMF), glycerol, and furfurals) [15, 16, 17], sacrificial regents (e.g., urea and ammonia) [18], and pollutants (e.g., sulfides and amines) (Figure 1a) [19, 20]. The electrochemical oxidative conversion of these substrates driven by their corresponding thermodynamic electrode equilibrium potentials replaced the kinetically sluggish OER [21], reducing substantially the electrolyzer voltage and overall energy input– but only once sufficient reaction rate, high selectivity, and stability are realized (Figure 1b). Among these alternative anodic oxidation reactions, the SOR (S^2−^ − 2e^−^ → S, *E*_0_ = 0.142 V vs. RHE, pH = 14) is considered to be very feasible.

Thus, we first use the techno‐economic modelling to guide process development of the SOR and evaluate the economic feasibility (Figure 1c,d; details in the Supporting Information). In this process, sulfion‐containing electrolytes (possibly derived from industrial sewage and off‐gas containing sulfide contaminants) are introduced into the SOR electrolyzer, where S^2−^ is electrochemically oxidized to elemental S along with the cathodic production of H_2_. After the key electrolysis step, a series of chemical processes (e.g., neutralization, condensation, crystallization et al) is taken to recover the valuable products—elemental S and sodium formate (HCOONa). Thus, from the simplified TEA, the industrial viability of this electrochemical system is governed by two trade‐off performance metrics: renewable electricity cost and the activity and stability of the catalyst. For instance, at a current density of 300 mA cm^−2^, the system could be economically attractive, provided that the renewable electricity cost is below $0.06 kWh^−1^. Moreover, a 500‐h catalyst stability reduces electrolyzer costs by 33.6% vs a 24‐h system. Thus, the SOR system holds even greater economic promise, if the current density and stability—determined by both the catalyst and the process methodology—would be further increased.

### 2.2 Sc doping Optimizes Intermediate Adsorption on NiFe‐LDH Electrocatalyst

**FIGURE 2:**
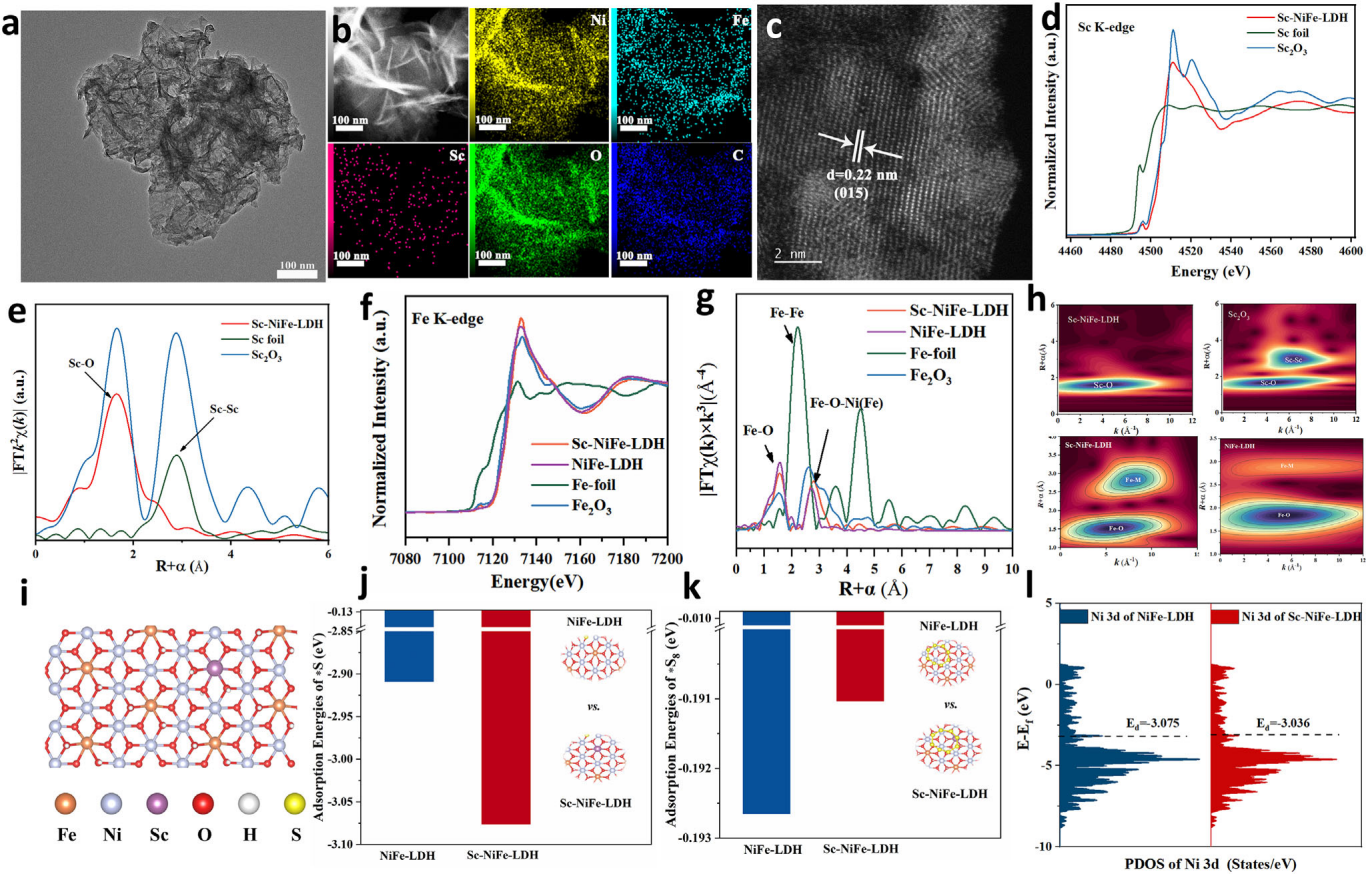
(a) TEM image; (b) AC HAADF‐STEM image of Sc‐NiFe LDH and the corresponding elemental mappings; (c) HRTEM; (d) Normalized Sc K‐edge XANES and (e) Fourier transform of the EXAFS spectra of Sc‐NiFe‐LDH and the references (Sc foil, Sc_2_O_3_); (f) Normalized Fe K‐edge XANES and (g) Fourier transform of the EXAFS spectra of Sc‐NiFe‐LDH and the references (Fe foil, Fe_2_O_3_, NiFe‐LDH); (h) WT‐EXAFS contour plots of the Sc_2_O_3_, NiFe‐LDH, Sc‐NiFe‐LDH; (i) Model of Sc‐NiFe‐LDH; DFT calculated adsorption energies (j) S^2−^; (k) S_8_; (l) The projected density of states (PDOS).

Scandium (Sc) doping in NiFe‐LDH was experimentally carried out via a hydrothermal method, aiming to regulate the intermediate adsorption and generate new active sites for improved activity and performance [22]. Sc was selected as a dopant for NiFe‐LDH, because its slightly larger atomic radius than Ni and Fe, to modulate the electronic micro‐environment of the active site, and its stable ionic radius (Sc^3+^) is compatible with those of Ni^2+^ and Fe^3+^, permitting solutioning incorporation in the lattice without phase instability, to facilitate electronic transfer in catalysis, as further analysed later. Particularly, the redox‐inert 3d° configuration and Lewis acidity of Sc^3+^ allow electronic modulation of neighboring Ni active sites, to further enhance the catalytic performance. Taking the 5% Sc doped NiFe‐LDH (Sc‐NiFe‐LDH), which showed the best performance, as an example, transmission electron microscopy (TEM) images reveal that the Sc‐NiFe‐LDH exhibits a nanoflower‐like morphology composing of thin, interconnected nanosheets (Figure 2a), which is further supported by the SEM observations (Figure S1). AC HAADF‐STEM image of Sc‐NiFe LDH and corresponding elemental mappings in Figure 2b confirms that Ni, Fe, Sc, O, and C are uniformly distributed throughout the sample. High‐resolution transmission electron microscopy (HRTEM) reveals clear lattice fringes with an interplanar spacing of approximately 0.22 nm (Figure 2c), which can be assigned to the (015) plane of Sc–NiFe–LDH. This value is slightly larger than that of pristine NiFe–LDH (0.21 nm), indicating a slight lattice expansion induced by Sc doping [23]. The powder X‐ray diffraction (XRD) patterns of both Sc‐NiFe‐LDH and NiFe‐LDH are presented in Figure S2. The positions of the diffraction peaks are in good agreement with the characteristic lattice parameters of NiFe‐LDH (PDF#40‐0215) [24]. Sc‐NiFe‐LDH exhibits narrower diffraction peaks than NiFe‐LDH, suggesting its enhanced crystallinity and increased grain size, which would reduce grain boundary resistance and facilitate more efficient charge transport [25]. Additionally, control samples including Ni(OH)_2_ and iron hydroxy carbonate (Fe_6_(OH)_12_CO_3_) were also prepared via a similar synthetic route, and characterized (Figure S3).

To ascertain the chemical state and coordination environment of the incorporated Sc dopant, X‐ray absorption near‐edge structure (XANES) and extended X‐ray absorption fine structure (EXAFS) measurements were conducted. Compared with those of Sc foil and Sc_2_O_3,_ the Sc K‐edge XANES spectra show that the absorption edge of Sc–NiFe–LDH shifts to higher energy (Figure 2d); indicative of a higher oxidation state of Sc in the doped Sc–NiFe–LDH. Fourier‐transformed EXAFS spectra at the Sc K‐edge (Figure 2e) further corroborate this conclusion. The spectrum of Sc–NiFe–LDH exhibits a prominent peak at approximately 1.8 Å, which can be assigned to the Sc–O scattering path. Notably, no discernible peak is observed at ∼2.8 Å, the characteristic Sc–Sc scattering path that are typically presented in Sc foil and Sc_2_O_3_. The absence of Sc–Sc coordination in Sc–NiFe–LDH strongly suggests that Sc atoms are atomically dispersed within the NiFe–LDH lattice (Figure 2h). The Fe K‐edge of Sc‐NiFe‐LDH shown in Figure 2f shifts slightly to higher energy, indicating an increased Fe valence state upon Sc doping. Figure 2g shows an extension of the Fe−O−Ni(Fe) (second shell scattering) bond length. Compared to NiFe‐LDH, the WT‐EXAFS analysis of Sc‐NiFe‐LDH reveals a ∼0.1 Å upshift for Fe−O−Ni(Fe) in R‐space (Figure 2h), further confirming the elongated Fe─O─Ni(Fe) bond. These changes in Fe−O−Ni(Fe) bonds, induced by Sc doping, lead to lattice expansion and influence the adsorption energy [26]. In addition, Figure S4a shows a higher oxidation state of Ni in Sc‐NiFe‐LDH than that in NiO. Moreover, Figure S4b and the EXAFS fittings shown in Table S1 also reveal that Sc doping elongates the Ni─O bond; indicative of the Sc incorporation caused the lattice expansion and influence the Ni valence state and the related adsorption energy (vide infra) [27].

To further elucidate the influence of Sc doping on the electronic structure and adsorption behavior, density functional theory (DFT) calculations were performed to evaluate the adsorption energies of Sc‐NiFe‐LDH and NiFe‐LDH for S^2−^ and S_8_ (Sulfur octamer) (Figure 2i, Figures S5 and S6). As shown in Figure 2j, the adsorption energies of S^2−^ on NiFe‐LDH and Sc‐NiFe‐LDH are −2.90 and −3.07 eV, respectively, indicating the potential enhanced SOR performance over Sc‐NiFe‐LDH. Furthermore, Sc‐NiFe‐LDH exhibits a lower adsorption energy for S_8_, demonstrating a faster desorption of the sulfur product (Figure 2k), which prevents the blockage of the active sites [28]. Meanwhile, the projected density of states (PDOS) and corresponding *d*‐band centers (*E*_d_) were further explored. As shown in Figure 2l, the *E*_d_ values of NiFe‐LDH and Sc–NiFe‐LDH are −3.075 and −3.036 eV, respectively. This result suggests that the upshift in the *d*‐band center upon Sc incorporation enhanced electronic interaction between Ni sites and *S intermediates, thereby facilitating their adsorptions and promoting the catalytic activity [8].

### 2.3 Catalyst Passivation Arises from Sulfur Poisoning in Continuous Electrolysis

**FIGURE 3:**
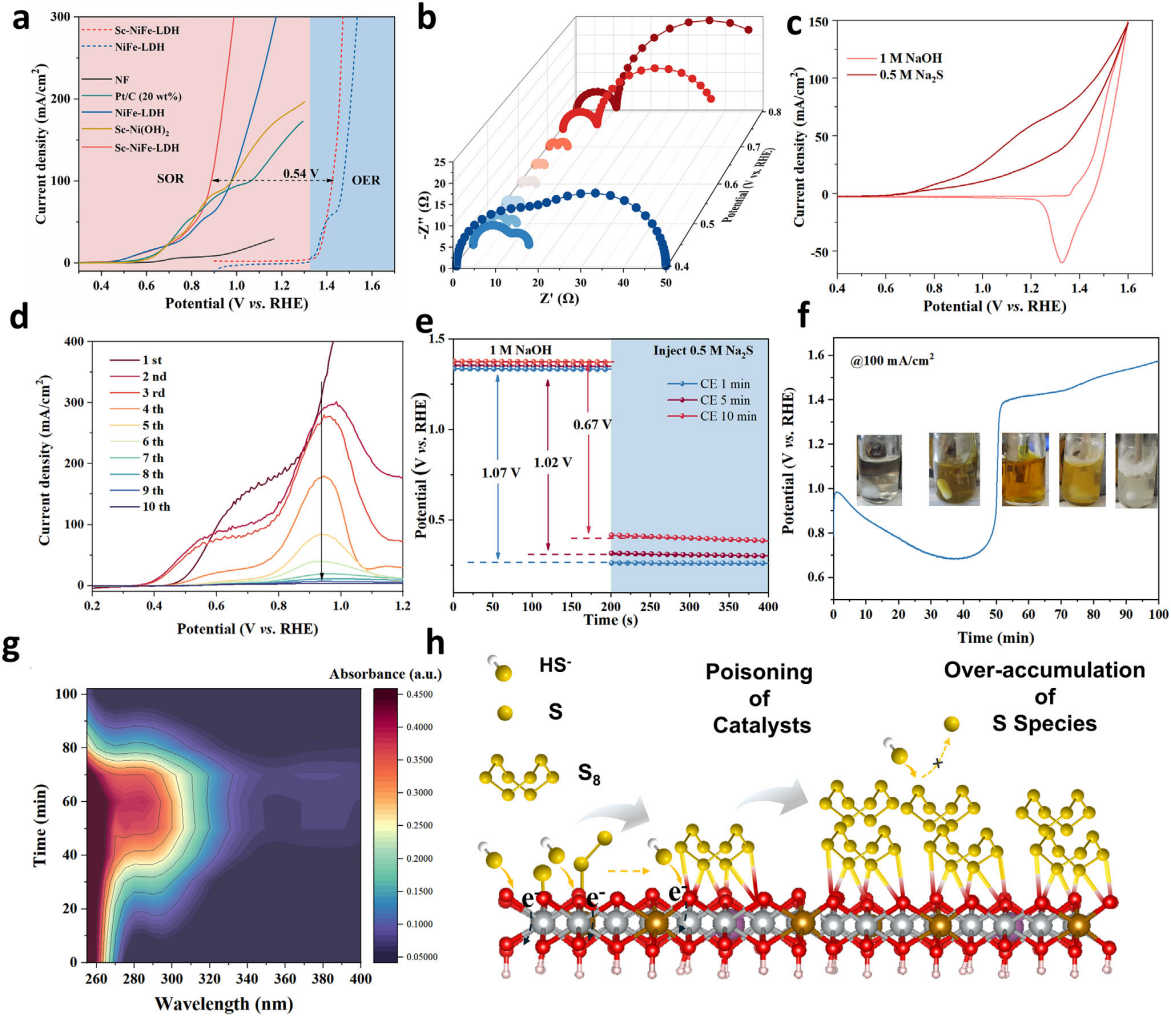
(a) LSV curves of SOR for Sc‐NiFe‐LDH and control electrodes. (b) Nyquist plots at various potentials; (c) CV curves of Sc‐NiFe‐LDH in 1 m NaOH with and without 0.5 m Na_2_S; (d) LSV curves of Sc‐NiFe‐LDH electrode in 1 m NaOH with Na_2_S at different scan times; (e) Open‐circuit potential curves of Sc‐NiFe‐LDH after CE with different times in NaOH, with Na_2_S being injected subsequently. (f) Chronopotentiometric curves of Sc‐NiFe‐LDH at 100 mA cm^−2^; (g) UV–vis spectra during SOR; (h) Schematic illustration of CE‐SOR over Sc‐NiFe‐LDH.

The SOR performance of the Sc‐NiFe‐LDH catalyst is evaluated in 1.0 m NaOH with different S^2−^concentrations, allowing the identification of the best condition at the S^2−^concentration of 1 m in terms of the LSV measured‐current density. Furthermore, 5% Sc doping was experimentally confirmed to be the optimal doping amount (Figure S7a,b). As shown in Figure 3a, the initial oxidation of S^2−^ occurs at a low potential of 0.45 V vs. RHE_;_ a 0.9 V positive shift relative to OER, and the required potentials to drive 100 and 300 mA cm^−2^ were only 0.88 and 0.99 V vs. RHE, respectively, suggesting a much more kinetically favorable SOR than OER. Sc‐NiFe‐LDH exhibits superior catalytic activity relative to the benchmarks (NF, Pt/C, Sc–Ni(OH)_2,_ and NiFe‐LDH). Notably, in comparison with NiFe‐LDH, it demonstrates a lower Tafel slope (13.5 mV dec^−1^), reduced charge transfer resistance, and an increased electrochemical surface area (ECSA) (Figures S8–S11). Moreover, concerning the single components control samples, Ni(OH)_2_ and iron hydroxy carbonate, we noticed that the higher current response and removal rates were generally achieved for Ni(OH)_2_ than that of iron hydroxy carbonate, indicating primary that Ni could be the dominant active center at the SOR (Figure S12).

Operando electrochemical impedance spectroscopy (EIS) was further carried out to understand the relationship between internal oxidation and electrode interface reactions during OER and SOR (Figure 3b, Figures S13 and S14). In the Bode plot, the high‐frequency region (10^1^ – 10^5 ^Hz) suggests the oxidation of the catalyst surface, while at the low‐frequency region (10^−2^ – 10^0^ Hz), it is associated with the heterogeneous charge distribution resulting from the electrochemical reactions [29, 30, 31]. In the absence of S^2−^ (Figure S13a), although the phase angle in the low‐frequency region decreased as the potential increased from 0.3 to 0.55 V vs. RHE suggest the accumulation of OH^−^ on the catalyst, the phase angles almost have no change in high‐frequency at the whole voltage, indicating that the structure of the catalyst has not transformed [32]. These results align well with the low current density observed in 0.3–0.8 V at LSV. Upon adding 0.5 m S^2−^ (Figure S13b), the Bode plots of the NiFe‐LDH electrode in the low‐frequency region exhibit a sharp decrease in phase angle compared to the OER, suggesting a rapid discharging process at the interface of S^2−^. On the other hand, the new peak emergence at 0.5 V vs. RHE in the low‐frequency region suggests the formation of S_n_^2−^, which is accompanied by the sharp increased current density. Moreover, the observed decrease in peak intensity at high frequency, accompanied by a shift toward lower frequency, indicates that the catalyst surface underwent structural evolution during SOR.

To further decouple these overlapping electrochemical processes, DRT (Distribution of Relaxation Times) analysis was performed (Figure S13c,d). The results reveal additional relaxation processes in the relaxation time range of *τ* ≈ 10^−1^–10^1^ s upon the introduction of sulfide species, which are absent in sulfide‐free alkaline electrolytes. These relaxation processes are the sulfide adsorption, oxidation of sulfur species, and the formation of sulfur‐containing intermediates during SOR. Notably, the intensified contributions in this relaxation time range at elevated potentials indicate increasingly limited mass transport and accumulation of sulfide‐derived species at the electrode/electrolyte interface. Similarly, the Nyquist plots (Figure 3b) showed the decreased semicircle diameters, indicating the lower charge‐transfer resistance (*R*_ct_) (Figure S14) and faster SOR reaction kinetics when the potential increased from 0.3 to 0.8 V vs. RHE [33]. At cyclic voltammetry curves shown in Figure 3c, Sc‐NiFe‐LDH exhibits obvious anodic and cathodic peaks at 1.38 and 1.33 V vs. RHE, respectively, corresponding to the redox couple of Ni^2+^/Ni^3+^ [34]. In the presence of Na_2_S, although the anodic current rises sharply with the increasing potential, the cathodic peak disappears; indicative of that the SOR intermediates/products in the forward sweep do not undergo reduction in the reverse sweep, and they have accumulated and therefore passivated the electrode surface [33, 35]. In contrast, in the LSV of the NiFe‐LDH with S^2−^ (Figure 3d), it shows the onset potential at 0.45 V vs. RHE, and the current density increases to 100 mA cm^−2^ at 0.88 V vs. RHE_,_ suggesting the electrochemical oxidation of S^2−^ into S_n_^2−^. Upon increasing the scanning times, the onset potential continuously positive shifts to 0.40 V vs. RHE. However, the anodic current density gradually decreases. This is because the accumulation of sulfur species on the electrode surface, which induces the passivation of the Sc‐NiFe‐LDH that diminishes its electrochemical activity. This phenomenon also accounts for the disappearance of the cathodic peak in the CV curve. Furthermore, the catalyst passivation was not only due to the formation of poisonous species, but also the changes in the catalyst structure restrict further the substrate adsorption, leading to a decrease in current during prolonged electrolysis. Concerning this, we further conducted the open‐circuit potential (OCP) test to evaluate Na_2_S adsorption at 0.7 V vs. RHE (Figure 3e).

Upon S^2−^ injection, the OCP decreased from 1.07 to 0.67 V vs. RHE over time. This indicates the weakened S^2−^ adsorption, likely caused by excessive sulfur accumulation, which reduced catalyst accessibility and hindered further adsorption. We next performed a chronopotentiometry experiment at 100 mA cm^−2^ and took photographs periodically to visualize the deactivation process by direct observing the color change of the electrolyte (Figure 3f). As shown, the sharp voltage rise during SOR for Sc‐NiFe‐LDH suggests surface passivation by insulating sulfur species; this rises the overpotential and increases the energy consumption by 40% within 10 min, suggesting the unsustainability of CE modes [36]. Meanwhile, the color of the anolyte gradually changed from colorless to orange and eventually to transparent, along with the milky‐white particles floating on the surface (Figure 3f inset). We then conducted UV–vis spectrophotometric analysis to track the transformations of sulfur‐containing intermediates (Figure 3g). The appearance of absorption peaks at approximately 260 and 300 nm corresponding to polysulfide ions indicated their formations as oxidation products [20, 37]. The intensities of these peaks increased significantly in the first 60 min, indicating the accumulation of polysulfide ions and deepening the electrolyte color. However, these peaks gradually diminished and eventually disappeared after 90 min of electrolysis, and finally the color of the electrolyte become milky white. This is attributed to the formation of sulfur (S^0^, 0‐valence sulfur), which initially exists as fine colloidal particles and imparts a transparent yellow hue to the solution. As electrolysis progresses, the concentration of sulfur colloids increases, intensifying the color to orange–yellow. Finally, the colloidal sulfur aggregates into larger particles, resulting in a milky‐white suspension [38]. Since UV–vis spectroscopy cannot detect S^0^, the disappearance of the S_n_^2−^ peaks reflect the transformation from soluble polysulfide to colloidal sulfur. XRD confirmed that the powder collected from the electrode surface was sulfur (Figure S15). Therefore, conventional CE requires intermittent stops to remove sulfur from the electrode surface, typically via mechanical electrode rotation or solvent extraction [10]. The slow desorption rate of intermediates leads to the deposition of S^0^ on the anode surface, the high resistivity of which causes progressive depletion of active species near the electrode interface. Consequently, sulfur poisoning of catalysts during SOR led to a decline in current density and sluggish oxidation kinetics (Figure 3h).

### 2.4 Pulsed Electrolysis Prevents Passivation by Dynamic Surface Refreshing and the Mechanism

**FIGURE 4:**
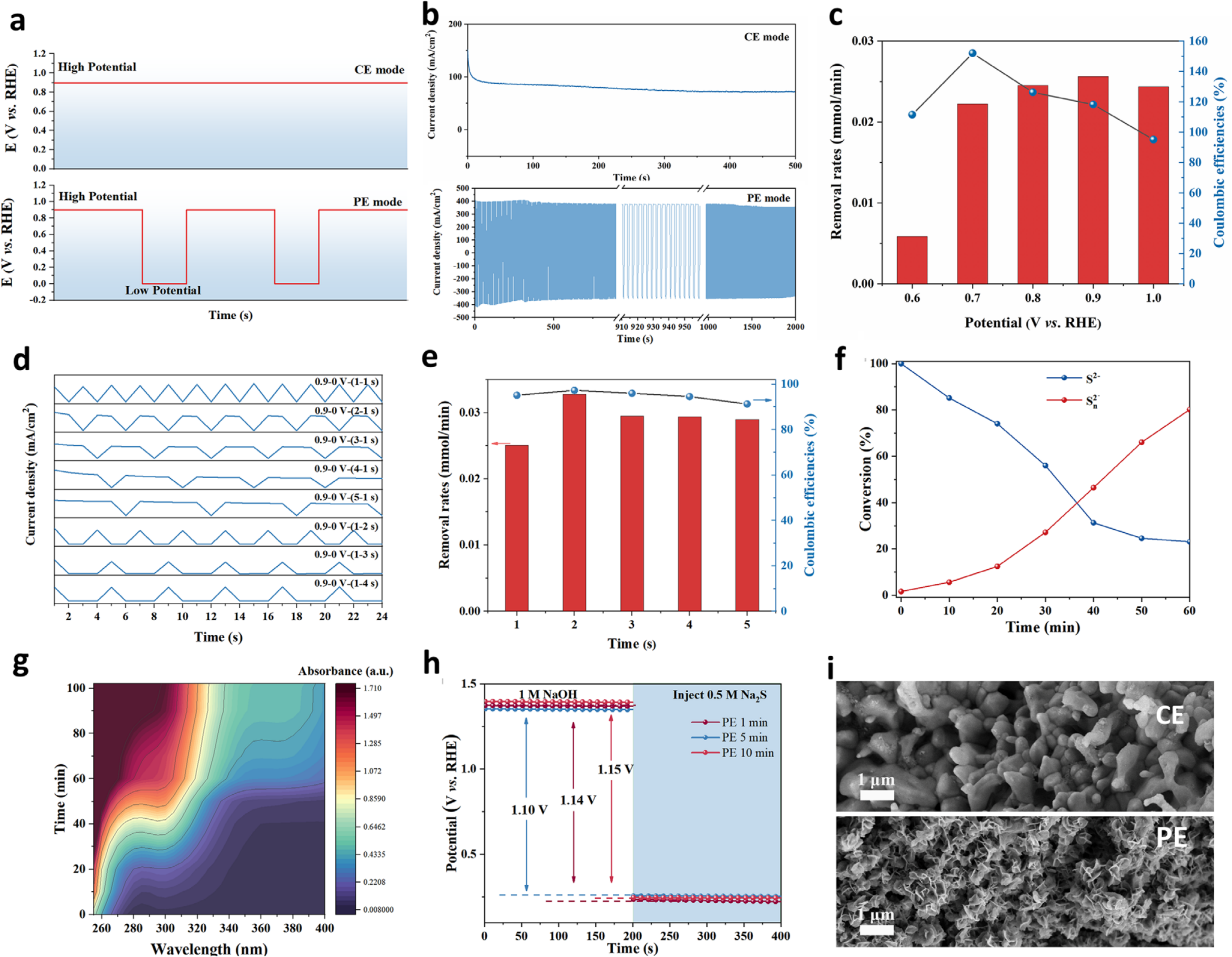
(a) Output potentials and (b) Chronoamperometric curves at CE and PE modes. (c) Removal rates of S^2−^ and coulombic efficiencies under different potentials at CE model; (d) Output potentials of a pulsed square wave with different high and low potential duration times; (e) Removal rates of S^2−^ and coulombic efficiencies at PE model (*E*_0.9_ = 1–5 s, *E*_0_ = 1 s); (f) Concentration changes of S^2−^ and S_n_^2−^ at PE mode (*E*_0.9_ = 2 s, *E*_0_ = 1 s); (g) UV–vis spectra during SOR; (h) Open‐circuit potential curves of Sc‐NiFe‐LDH at PE model; (i) SEM images of the catalysts after reaction at either CE or PE model.

To address those issues, we then sought to use PE technology to reduces sulfide‐related intermediates/products absorptions and accumulations, thereby exposing active sites and preventing the poison of Sc‐NiFe‐LDH catalyst. To validate these, we first conducted SOR at either CE or PE modes using the potential square‐wave outputs (Figure 4a) [39]. At CE mode, a constant high oxidation potential was applied. In contrast, PE mode repeatedly alternated between periods of high oxidation potential and low OCP. Notably, a significant reverse charging current was rapidly generated upon pulsing from high potential to low OCP, facilitating the charging of the surface double layer on the electrocatalyst. Thus, we compared the performances in CE and PE modes. As shown in Figure 4b, in CE mode, the current density decreased rapidly even at the initial stage. In contrast, PE mode maintained a high current density throughout the process, exhibiting symmetrical and periodic fluctuations; those indicates the excellent stability and reversibility (Figure S16).

During electrolysis, the SOR proceeds via two simultaneous pathways in alkaline solution: (I) electrochemical conversion of soluble sulfide ions to insoluble elemental sulfur (8 S^2−^ (aq) → S_8_(s) + 16e^−^); and (ii) chemical dissolution of elemental sulfur back into polysulfide species (e.g., (x–1) S(s) + S^2−^ (aq) → S_x_^2−^ (aq)), which is the reverse of the electrochemical pathway [40]. The electrocatalytic performance of Sc‐NiFe‐LDH was evaluated and compared at CE and PE modes in terms of sulfide removal rates and coulombic efficiencies (Figure 4c,d, Figure S17) [10]. At CE mode, sulfide removal rates increased progressively with the applied potential, reaching 0.025 mmol min^−1^ at 0.9 V vs. RHE after 3600 s. However, coulombic efficiencies higher than 100% were observed between 0.6 and 0.9 V, which is attributed to the formation of polysulfides at the electrode–electrolyte interface via a chemical reaction between the already deposited S^0^ and the dissolved sulfide; both processes are thermodynamically and kinetically favorable at high pH [41]. Higher potential favors the faster electrochemical route for sulfur deposition; thus, when the applied potential reached 1.0 V vs. RHE, the coulombic efficiency decreased to 95.1%. However, the generated S^0^ strongly interacted with catalyst surfaces, leading to rapid catalyst deactivation and anode passivation. The conclusion is further supported by the SEM imaged morphological changes in the catalyst after electrolysis at different potentials (Figure S18a–c).

To alleviate anode passivation, SOR should be carried out at low current densities; however, this will compromise electrolysis efficiency. In contrast, the PE mode enhances mass transport and facilitates the chemical dissolution of deposited sulfur, thereby restoring the dynamic balance between sulfur dissolution and deposition even at high potentials. To investigate the effect of the PE mode, eight square waveforms with different durations of high and low potentials were designed (Figure 4d). When PE was applied (*E*_0.9_ = 1–5 s, *E*_0_ = 1 s, where *E*_0.9_ and *E*_0_ denote the applied potentials of 0.9 and 0 V vs. RHE, respectively) during SOR, the sulfide removal rate increased from 0.030 mmol min^−1^ under CE (0.9 V) to 0.032 mmol min^−1^. Coulombic efficiencies at PE mode remained below 100%, with a maximum of 99.84% observed at *E*_0.9_ = 2 s, *E*_0_ = 1 s (Figure 4e). As expected, when the pulse configuration was reversed (*E*_0.9_ = 1 s, *E*_0_ = 1–5 s), the coulombic efficiency exceeded 100%. This is because the prolonged open‐circuit period promoted the chemical dissolution of elemental sulfur (Figure S19) [41]. Figure 4f shows the concentration changes of S^2−^ and S_2_^2−^ during the reaction, with a rapid sulfide removal rate of 0.032 mmol min^−1^ and a high efficiency of 77% achieved after 60 min of electrolysis. The corresponding UV–vis spectra showing the increase of S_n_^2−^ in the anolyte during SOR are markedly different from those obtained under chronopotentiometric electrolysis at 100 mA cm^−2^, indicating the continuous formation of S_n_^2−^ throughout the SOR process (Figure 4g, Figure S20) [42]. This demonstrates that the PE strategy effectively prevents sulfur‐induced catalyst passivation and enhances the overall kinetics of SOR (Figure 3g, Figure S18 d‐). The OCP analysis at PE mode showed a potential increase from 1.10 to 1.15 V vs. RHE (Figure 4h), indicating enhanced S^2−^ adsorption and implying that the catalyst was activated [33]. We further evaluated the performance of the post‐catalyst after SOR at either CE or PE mode by conducting again the CV and LSV measurements. While both CV and LSV results suggested that the post‐reaction Sc‐NiFe‐LDH catalysts with significant decreased electrochemical performance in SOR at the CE model, the reaction using the same catalyst conducted at PE mode exhibited improved performance (Figure S21). Moreover, SEM images of the post‐reaction catalysts at CE and PE modes clearly show distinct morphological differences (Figure 4i). In CE mode, the catalyst surface exhibited pronounced protrusions due to the sulfur deposition (Figure S15), confirming that excessive sulfur accumulation caused the catalyst deactivation. In contrast, at PE mode, the original nanosheet structure became thinner compared to the as‐synthesized state, suggesting increased exposure of active sites and enabling sustained electrolysis.

**FIGURE 5:**
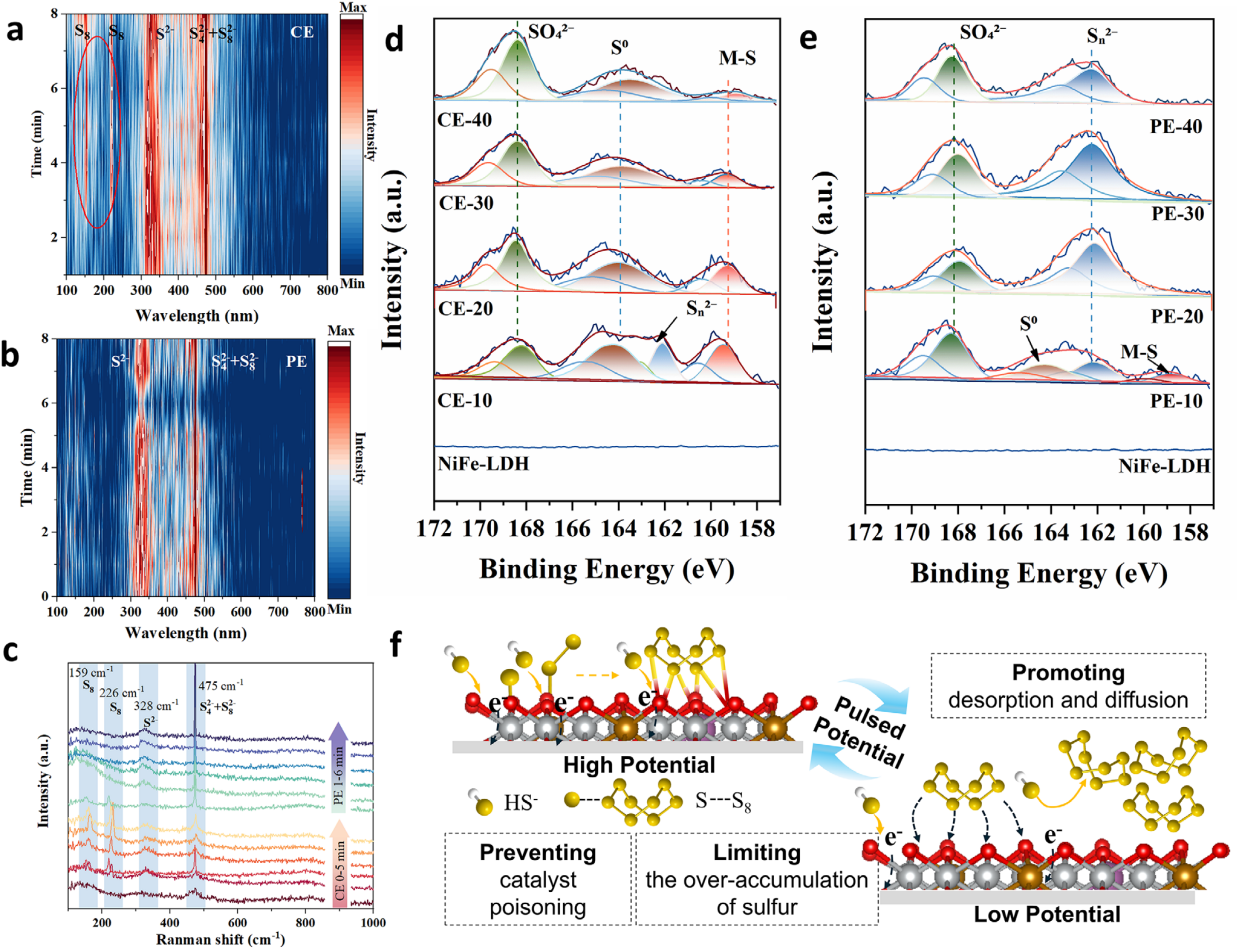
In situ Raman spectra collected at (a) CE mode; (b) PE mode; (c) CE for 5 min then PE for 6 min; ex situ XPS spectroscopy of Sc‐NiFe‐LDH electrode for SOR during the time course of 10 to 40 min at (d) CE mode; (e) PE mode; (f) Schematic illustration of the mechanism of Sc‐NiFe‐LDH catalyzed SOR at PE mode.

We then employed in situ Raman and ex situ X‐ray photoelectron spectroscopy (XPS) at CE and PE modes to identify the reaction intermediates during SOR. As shown in Figure 5a, after electrocatalysis at CE mode for 3 min, four prominent Raman signals were observed at 159, 226, 328, and 475 cm^−1^, corresponding, respectively, to S_8_, S_8_, S^2−^, and S_4_^2−^ + S_8_^2−^ species on the catalyst surface [7, 40]. This indicates that solid‐state sulfur, in addition to intermediate sulfur species, was rapidly formed on the catalyst surface within a short time. In contrast, the characteristic peaks of solid‐state sulfur at 159 and 226 cm^−1^ were not detected within 8 min at PE mode (Figure 5b), indicating that the PE model effectively suppresses S_8_ accumulation and electrode passivation, thereby sustaining the continuous formation of soluble polysulfide intermediates (e.g., S_4_^2−^/ S_8_^2−^). To better understand how the PE mode influences the catalyst structure during the reaction, in situ Raman experiments were conducted in which a constant voltage was initially applied to the electrode, followed by a pulsed voltage. As shown in Figure 5c, after 5 min of electrocatalysis at CE mode, the Raman signals corresponding to S_8_ at 159 and 226 cm^−1^ increased markedly, indicating the substantial accumulation of elemental sulfur on the catalyst surface [43]. However, upon switching to PE mode, these signals gradually diminished, suggesting that adsorbed elemental sulfur was desorbed from the catalyst surface. In addition, we also conducted in situ Raman experiments on Sc‐NiFe‐LDH catalyzed SOR at CE mode under different applied potentials; results show that elemental sulfur was rapidly adsorbed on the catalyst surface even at a low potential of 0.7 V vs. RHE (Figure S22). Thus, reaction at the PE mode effectively promoted the desorption of poisoning sulfur species and regenerated clean active sites on the metal catalyst surface for continuous SOR with high performances (vide supra).

To further reveal the evolution of sulfur species and their interactions with the active sites, we next performed Ex situ XPS analysis at both CE and PE modes at different reaction times (10–40 min). The S 2p XPS spectra then provided valuable insights into sulfur speciation and the underlying mechanism of catalyst deactivation. In continuous electrocatalysis at CE mode (Figure 5d), the spectra exhibit a peak at ∼164.4 eV corresponding to S^0^, and another at ∼168.3 eV attributed to sulfate species (SO_4_^2−^), whereas signals corresponding to lower‐valence sulfur species (S_n_^2−^, ∼162.2 eV) are detected only after 10 min of CE, suggesting that S_n_^2−^ is a short‐lived intermediate formed during the initial stage of CE [44, 45, 46]. Notably, the pronounced peak at 159.8 eV, the characteristic of M─S bonding [47], indicates the strong interactions between sulfur and the metal sites, which, on the one hand promote the oxidation of intermediate species at the catalyst surface; on the other hand, it also accelerates the accumulation of S^0^. However, the absence of the S_n_^2−^ peak at CE mode for 20–40 min suggests that polysulfides cannot be continuously oxidized to S_8_. In contrast, at PE mode (Figure 5e), the S 2p spectra display sustained signals for S_n_^2−^ and trace amounts of S^0^ and Metal–S; those are observed only at PE‐10. Those observations suggest that the PE mode disrupts the metal–S interactions, facilitating the continuous generation and replenishment of soluble polysulfide intermediates (S_4_^2−^/S_8_^2−^), and preventing the rapid accumulation of S^0^ and the passivation of metal active sites. Consequently, the intermittent operation in PE mode mitigates electrode passivation and maintains catalytic activity by preventing the localized accumulation of sulfur species that were typically observed at CE mode.

In contrast, the Ni 2p spectra (Figure S23) show a dynamic evolution between Ni^3+^ and Ni^2+^ states during SOR, suggesting the influence of the oxidative environment on the electronic state of the metal centers in the catalyst [48]. At CE mode (Figure S23a), the Ni 2p spectra show a progressive increase in the Ni^3+^ intensity (857.3 eV). This suggests that continuous sulfur oxidation promotes the oxidation of Ni^2+^ to Ni^3+^, and the Ni^3+^/Ni^2+^ ratio increases rapidly with prolonged reaction time. On the other hand, at PE mode (Figures S23b and S25a), the Ni^3+^/Ni^2+^ ratio remains almost no change throughout the reaction. This suggests that the pulsed potential in PE mode effectively mitigates over‐oxidation and prevent the detrimental surface reconstruction of the catalyst. In the Fe 2p spectra (Figure S24), peaks at 712.7 and 707.6 eV were assigned to Fe^3+^ and Fe^2+^, respectively [49, 50, 51]. In contrast to Ni, the Fe^3+^/Fe^2+^ ratio is not changed at both CE and PE modes (Figure S25b), further suggesting that Ni serves as the primary active site for SOR. Thus, it is suggesting that sulfur atoms are strongly bound to Ni sites, progressively blocking them and causing the catalyst passivation. As a result, it hinders the oxidation of polysulfide intermediates, increases the overpotential, and slows down the reaction kinetics of SOR. In contrast, in the PE mode, the disruption of Ni–S effectively exposes Ni active sites, facilitating the desorption and diffusion of S_n_^2−^ for continuous oxidation. This arises from the alternating potentials at PE, where high potentials partially oxidize sulfur species, and low potentials promote their desorption. Such dynamic regulation in PE mode prevents sulfur accumulation and catalyst poisoning, thereby preserving active sites, suppressing passivation, and promoting reaction kinetics (Figure 5f).

### 2.5 Scaled‐up SOR Electrolyzer Shows High Efficiency and Stability

**FIGURE 6:**
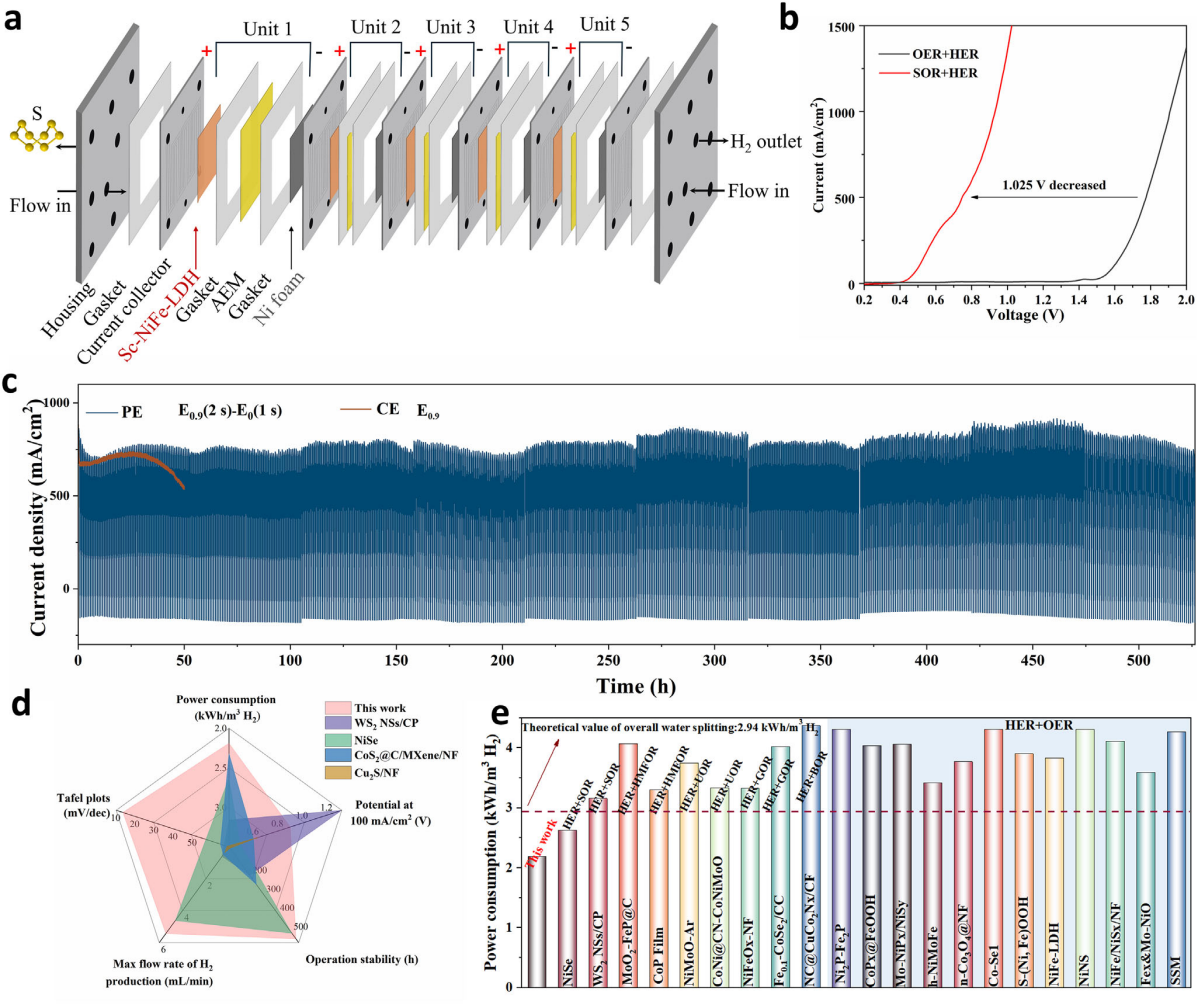
(a) Schematic diagram of stacked MEA system consisting of five stacked modules; (b) LSV curves of Sc‐NiFe‐LDH in 1 L of 1 m NaOH with or without 1 m Na_2_S in MEA; (c) Catalyst's durability tests at PE and CE mode (replacing fresh electrolyte every 52 h); (d) Comparison of the catalytic performances of Sc‐NiFe‐LDH with the state‐of‐the‐art electrocatalysts; (e) Comparisons of the power consumptions between Sc‐NiFe‐LDH and literature‐reported multi‐functional electrocatalysts.

To probe the effectiveness of such a system in a more practical scenario, we assembled a stacked flow electrolyzer consisting of five units with a total active area of 20 cm^2^ (Figure 6a,b). The anode and cathode catalysts were symmetrically placed on the respective sides of the current collectors (Figure 6a). A mixed electrolyte of 1 m NaOH and 1 m Na_2_S was circulated through the flow reactor at a rate of 5 mL min^−1^ using a peristaltic pump. As shown in the LSV curves (Figure 6b), the SOR requires only 0.56 and 0.75 V to reach currents of 200 and 500 mA cm^−2^, respectively. In comparison with the conventional alkaline water electrolysis (HER + OER), the HER + SOR system substantial reduces the required voltage (e.g., 0.75 V compared to 1.77 V at 500 mA cm^−2^), showing the high energy efficiency.

We next conducted the stability test at both PE mode (*E*_0.9_ for 2 s, *E*_0_ for 1 s) and CE mode at a constant voltage of 0.9 V (Figure 6c). The PE mode exhibited exceptional durability. A slight decrease in current density was observed as the S_x_^2−^ concentration in the electrolyte gradually decreased. Nevertheless, the current density remained stable even after ten electrolyte replacements, sustaining catalytic performance for over 500 h; the performance significantly outperformed the reaction operated in CE mode (25 h). Hydrogen was produced at a high rate of 325.9 mL h^−1^, with a remarkably low energy consumption of 2.19 kWh m^−3^ H_2_; a 57.7% reduction in electricity cost compared to conventional alkaline water electrolysis (5.18 kWh m^−3^) (Figure S26). This performance surpasses the previously reported SOR and water‐splitting electrolyzers for hydrogen production (Figure 6d,e; Tables S2 and S3) [6].

### 2.6 Integrating Seawater Electrolysis and Formic Acid Upgrading for Sustainable H_2_ Production and Sulfide Valorization

**FIGURE 7:**
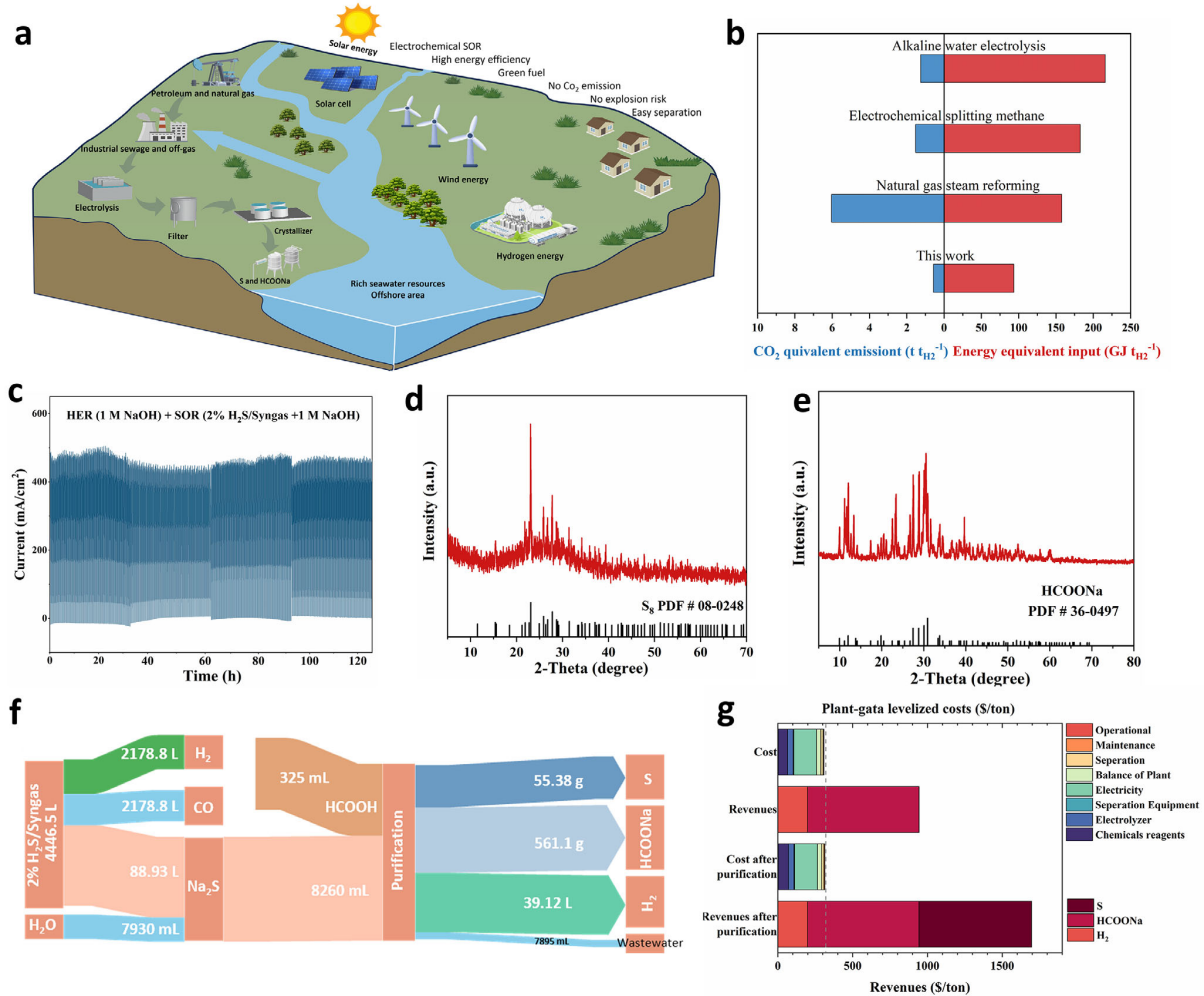
(a) Schematic illustration of the integrated process of sulfide contaminant recycling, sulfur production, and purification; (b) Comparison of a hybrid seawater‐SOR electrolyzer system with different hydrogen production techniques in energy equivalent input and CO_2_ equivalent emission; (c) Durability of Sc‐NiFe‐LDH for the electrochemical removal of H_2_S from industrial syngas (2% H_2_S) in 1 m NaOH solution; (d) XRD of prepared S_8_; (e) XRD of obtained sodium formate; (f) Sankey diagram for the mass flow of industrial syngas upcycling; (g) SOR profit comparisons before and after sulfur purification.

This SOR strategy enables sustainable hydrogen sulfide valorization under mild conditions by coupling cathodic green hydrogen production with anodic conversion of sulfides into value‐added products, achieving net‐zero process emissions. To further cut operational costs, we, on the one hand, exploited the system's ultralow thermodynamic potential (0.142 V vs. RHE); on the other hand, we also implemented seawater as the electrolyte—experimentally achieving 100  mA cm^−2^ at only 0.66 V vs. RHE under ambient conditions (Figure S27), which further decreases to 0.40 V vs. RHE at 70°C. In this way, synergies with coastal ecosystems where abundant seawater, high‐density renewable energy inputs (offshore wind capacity factor: 100–200 W m^−2^; solar irradiance: 1200–1500 kWh m^−2^ a^−1^) [52], and the already existed chlor‐alkali infrastructure converge to provide NaOH co‐electrolyte would be created. Thus, integrating catalyst design and strategic resource utilization, a circular pathway for simultaneously advancing blue economy and pollution mitigation goals (e.g., upgrading sulfide pollutants) is established (Figure 7a). Furthermore, the hybrid seawater‐SOR electrolyzer system outperforms literature‐reported advanced hydrogen production methods (e.g., alkaline electrolysis, steam reforming of 2%H_2_S/Syngas, and electrochemical methane conversion Figure 7b, Table S4). Thus, it lowered energy input and CO_2_‐equivalent emissions, showcasing its energy efficiency and environmental sustainability.

As the proof‐of‐concept, we perform the SOR using the simulated industrial syngas (49% CO, 49% H_2,_ and 2% H_2_S) [53]. As shown in Figure S28, the SOR current response for the H_2_S/syngas mixture was markedly higher than that for either pure syngas or Ar, highlighting the effective oxidation of H_2_S catalyzed by Sc‐NiFe‐LDH. However, under identical conditions, the nearly identical LSV profiles for syngas and Ar suggest that syngas exhibits negligible electrochemical activity at the anode for its oxidation. Furthermore, a long‐term stability test at PE mode (*E*_0.9_ for 2 s and *E*_0_ for 1 s) demonstrated that the system, using 2% H_2_S/syngas and 1 m NaOH as the anolyte, remained stable for over 120 h (Figure 7c). These results highlight the excellent selectivity and long‐term operational stability of the system for H_2_S removal from industrial syngas, along with energy‐efficient hydrogen production. The anode materials after long‐term electrolysis were characterized by XRD, XPS, SEM–EDS, and TEM. No significant changes in morphology or crystal structure were observed for the catalyst after PE operation, whereas pronounced sulfur accumulation and surface reconstruction were detected after CE operation (as revealed by EDS mapping and HRTEM). These results further confirm the structural stability and anti‐passivation advantage of the PE strategy (Figures S29–S34).

Generally, elemental sulfur recovery is typically achieved via the acidification of the polysulfide anolyte with sulfuric acid (S_n_^2−^ + H^+^ → HS^−^ + (n‐1)/8 S_8_) [40]. However, this method consumes a substantial amount of acid, generates toxic H_2_S gas, and produces low‐cost raw elemental sulfur (0.22$ kg^−1^), limiting its practical profitability. To address these issues, we employed the bio‐derived formic acid as the acidifier for the electrolyte to enable sufficient sulfur precipitation, and to produce another value‐added product sodium formate followed by concentration and crystallization (Figure 7d,e). Sodium formate plays a significant role in agriculture, environmental protection, and the leather industry—especially as a feed additive for swine and poultry to lower gastrointestinal pH and inhibit harmful bacteria such as *E. coli, Salmonella spp*., etc—offering a considerably higher market value [54, 55]. In this way, as shown in the Sankey diagram of the mass flow analysis (Figure 7f), 2% H_2_S/syngas feedstock ultimately yielded 55.38 g of sulfur, 561.1 g sodium formate, and 39.12 L H_2_ as isolated pure products. After simple purification, high‐purity sulfur (99.5%, $140 kg^−1^), which is widely used in agriculture, pharmaceuticals, rubber, and the chemical industry, was obtained (Table S5). Thus, in this way, the TEA showed that the potential revenue increased markedly from $585.90 to $1294.71 tonne^−1^, indicating a promising and profitable prospect for industrial‐scale application (Figure 7g, Figures S34 and S35, Table S6). Taken together, integrating the hybrid seawater–SOR electrolyzer with renewable energy is expected to further enhance the economic viability and sustainability of the system. This work offers new insights into carbon‐neutral exploitation of abundant oceanic hydrogen resources.

## 3 Conclusions

In summary, by integrating PE with high‐performance Sc‐NiFe‐LDH, we overcome persistent electrocatalyst deactivation at SOR by controlling the dynamic surface microenvironment, where Sc^3+^ doping optimizes intermediate adsorption energies while pulsed operation accelerates sulfur desorption and mitigates active‐site oxidation. In this way it achieves 99.8% coulombic efficiency for sulfur recovery and hydrogen production at 2.19 kWh m^−3^ H_2_—57.7% lower than the conventional systems—with the stability >500 h. Crucially, substituting mineral acids with formic acid enables the circular co‐production of high‐purity sulfur (99.5%) and sodium formate, achieving a high profit to $1,294.71 per tonne after purification (121% increase). Our work thus establishes an industrially scalable platform for valorizing H_2_S‐rich waste streams, creating new economic paradigms for sustainable chemical manufacturing.

## Author Contributions

C.W. and Z.G. conceived the experiments and supervised the project. Y.W., Z.H., and Y.M. carried out the synthesis of electrodes, electrochemical measurements, part of the DFT calculations and analyzed data. Z.H., Y.M., and Y.P. carried out the synthesis, DFT calculations and analyzed data. All authors co‐wrote the paper. All authors discussed the results and contributed to the preparation of the manuscript.

## Conflicts of Interest

The authors declare no conflicts of interest.

## Supporting information




**Supporting File**: adma73096‐sup‐0001‐SuppMat.docx.

## Data Availability

The data that support the findings of this study are available from the corresponding author upon reasonable request.
