# Supplementary material for: Pulsed Electrolysis Prevents Sulfur Poisoning for Sustained Sulfide Valorization
Source: Adv Mater. 2026 Apr 17;38(28):e73096. doi: 10.1002/adma.73096 (PMC13181516; doi:10.1002/adma.73096)
Supplement: Supplementary file 1 — Supporting File: adma73096‐sup‐0001‐SuppMat.docx. [file ADMA-38-e73096-s001.docx]

**Supporting Information**

**Pulsed electrolysis prevents sulfur poisoning for sustained sulfide valorization**

Zhiyan Hou,^1^† Yangbo Ma,^2^† Yufeng Wu,^1^* Weijin Cao,^1^ Zhengxiao Guo,^2*^and Changlong Wang ^1^*

^1^ State Key Laboratory of Materials Low-Carbon Recycling, College of Materials Science and Engineering, Beijing University of Technology, Beijing 100124, China

^2^ Department of Chemistry, The University of Hong Kong, Hong Kong, SAR, China

† These authors contributed equally to this work.

* Corresponding authors.

E-mail addresses: [clwang1987@126.com](mailto:clwang1987@126.com); wuyufeng3r@126.com; zxguo@hku.hk

**This file includes:**

Supplementary Text

Figures S1 to S36

Tables S1 to S6

Outlook

References

**1. Materials and Methods**

**Chemicals**

All solvents and chemicals were used as purchased without further purifications. Chemicals: Sodium hydroxide (NaOH, 95%), sodium sulfide nonahydrate (Na_2_S·9H_2_O, 99%) were acquired from Adamas-beta, formic acid (CH_2_O_2_, 99.9%) were supplied by Shanghai Aladdin Bio-Chem Technology Co., Ltd. Ni(NO_3_)_2_·6H_2_O (Aladdin, 99.99%), Fe(NO_3_)_3_·9H_2_O (Aladdin, 99.99%), Sc(NO_3_)_3_·xH_2_O (Aladdin, 99.99%), anion-exchange membrane (Fumasep FAB-PK-130), Ni foam (Recemat BV, Netherlands). Prior to the synthesis, all the flasks were washed with HCl to remove any traces of metal residue. This was followed by washing with water and acetone and a final drying step at 80 °C overnight. NF has been cleaned sequentially with 3.0 M HCl, acetone, ethanol, and deionized water (DI water) before being used. Milli-Q water (18.25 MΩ) was used for all the syntheses and catalysis experiments.

**Characterizations**

The scanning electron microscopy test was conducted on ZEISS GeminiSEM 300. The X-ray photoelectron spectroscopy (XPS) measurements were recorded on a Thermo XPS ESCALAB 250Xi. The base pressure during the experiment in the analysis chamber was 3×10^-7^ mbar. To account charging effects, all spectra are referred to C 1s at 284.8 eV. The X-ray powder diffraction (XRD) characterization was conducted using a Bruker AXS D8 Advance diffractometer with a Cu-Kα source (1.54056 Å). UV-vis spectral analysis were obtained from a UV-2600. The transmission electron microscopy (high-resolution TEM (HRTEM)), and elemental mapping using a JEOL-JEM-2100F transmission electron microscope at an accelerating voltage of 200 kV.

Data reduction, data analysis, and EXAFS fitting were performed and analyzed with the Athena and Artemis programs of the Demeter data analysis packages (reference 1：B. Ravel and M. Newville, ATHENA, ARTEMIS, HEPHAESTUS: data analysis for X-ray absorption spectroscopy using IFEFFIT, Journal of Synchrotron Radiation 12, 537–541 (2005) ) that utilizes the FEFF6 program (reference 2：Zabinsky, S. I.; Rehr, J. J.; Ankudinov, A.; Albers, R. C.; Eller, M. J. Multiple-Scattering Calculations of X-Ray-Absorption Spectra. Phys. Rev. B 1995, 52 (4), 2995−3009.) to fit the EXAFS data. The energy calibration of the sample was conducted through standard Fe foil and Ni foil respectively, which as a reference was simultaneously measured. A linear function was subtracted from the pre-edge region, then the edge jump was normalized using Athena software. The χ(k) data were isolated by subtracting a smooth, third-order polynomial approximating the absorption background of an isolated atom. The k^3^-weighted χ(k) data were Fourier transformed after applying a HanFeng window function (Δk = 1.0). For EXAFS modeling, the global amplitude EXAFS (CN, R, σ^2^ and ΔE_0_) were obtained by nonlinear fitting, with least-squares refinement, of the EXAFS equation to the Fourier-transformed data in R-space, using Artemis software, EXAFS of the Fe foil and Ni foil was fitted respectively and the obtained amplitude reduction factor S_0_^2^ value (0.750 and 0.783) was set in the EXAFS analysis to determine the coordination numbers (CNs) in sample.

**Electrochemical measurements**

The oxygen evolution reaction (OER) and electrocatalytic oxidation of Na_2_S were tested using a Ivium-n-Stat (Ivium Technologies B.V., Netherlands) electrochemical workstation with the standard three-electrode system in an H-type electrochemical cell separated by an anion-exchange membrane (PK-130) at room temperature. The Sc-NiFe-LDH (~ 1 cm × 1 cm) were used as the working electrode (WE), a carbon rod was used as the counter electrode (CE), and a standard Hg/HgO electrode was used as the reference electrode (RE), and 1 m NaOH is used as an electrolyte solution. The OER were tested in 1 m NaOH electrolyte solution. The electro-oxidation of Na_2_S were tested in 5 mL 1 m NaOH with 0.1-1 m Na_2_S. Organic substrates were added only into the anode compartment. Stirring was applied for both the anode and cathode chamber at 300 rpm in the cathode and 700 rpm in the anode chamber. In a typical electrochemical experiment sequence, cyclic voltammetry (CVs) at a rate of 100 mV s^-1^ from 0.00 – 0.60 V vs. Hg/HgO for 20 cycles were measured to guarantee a stable electrode performance during the experiments. The potentials were converted to the reversible hydrogen electrode (RHE) through the Nernst equation: (E(RHE) = E(Hg/HgO) + 0.059 pH + 0.098 V). The scan rate for LSV was kept at 5 mV s^-1^. The electrochemical surface area (ECSA) was evaluated in terms of the double-layer capacitance (C_dl_). Cyclic voltammetry (CV) was performed in 1 m NaOH with 0.5 M Na_2_S at different scan rates of 10-50 mV s^-1^ in a potential window where no Faradaic process occurs (0.27 – 0.37 V vs. RHE). In situ Electrochemical impedance spectroscopy (EIS) measurements were conducted with a three-electrode system. The frequency ranged from 100000 to 0.01 Hz with an amplitude of 5 mV, and the potential applied ranged from 0.3 to 0.8 V (vs. RHE) with 0.05 V interval.

**In-situ Raman measurement：**

The in-situ Raman spectra were recorded using a confocal Raman microspectrometer (Renishaw, inVia-Reflex) equipped with an Ar laser (wavelength = 532 nm). The spectral range was 100-1000 cm^-1^. The laser power was set at 5 and the exposure time was 10 seconds. In-situ Raman spectra were recorded on the aforementioned Raman microscope under controlled potentials using the Chenhua CHI760 electrochemical workstation. The electrolytic cell, purchased from Shanghai Chu Xi Industrial Co., Ltd, was made of polytetrafluoroethylene (PTFE), and a thin round quartz glass plate was used as a protective cover for the objective. A Pt wire was used as the counter electrode, and an Ag/AgCl electrode was used as the reference electrode. The working electrode was inserted through the wall of the electrolytic cell to ensure that its plane was perpendicular to the incident laser.

**Synthesis of Sc-NiFe-LDH electrodes**

The The nickel foam (NF) was cut into slices (1 cm × 3 cm) and successively ultrasonicated in 3 M HCl, acetone, ethanol and water for 15 min, respectively. Sc-NiFe-LDH was prepared via a one-pot hydrothermal synthesis. In detail, Ni(NO_3_)_2_·6H_2_O (0.66 mmol), Fe(NO_3_)_3_·9H_2_O (0.3135 mmol), Sc(NO_3_)_3_·xH_2_O (0.0165 mmol), and urea (5 mmol) were dissolved in 30 mL of deionized water to form a clear solution. A pre-treated nickel foam (NF) substrate was immersed in this mixture, which was then transferred into a 50 mL Teflon-lined stainless-steel autoclave and heated at 120 °C for 10 h. After naturally cooling to room temperature, the obtained product was sequentially rinsed three times with deionized water and ethanol, followed by drying at 60 °C overnight. Additionally, Ni(OH)_2_ and Fe_6_(OH)_12_CO_3_ were synthesized using Ni(NO_3_)_2_·6H_2_O (1 mmol) and Fe(NO_3_)_3_·9H_2_O (1 mmol), respectively, with no other metal sources involved.

**Sulfide removal**

Measurement of S^2-^ removal efficiency in the 0.5 M Na_2_S + 1.0 M NaOH electrolyte. The UV spectrophotometric method was used to determine the S^2-^ concentration during the SOR. The calibration curve was obtained by measuring the UV-vis spectrum at λ = 230 nm with a standard sample. After the chronopotentiometry tests, the electrolytes were diluted 2000 times for the determination of S^2-^ concentration by UV–vis spectroscopy in the wavelength range of 200–300 nm. To analyze the S_2_^2-^/S_4_^2-^ species, the electrolytes were diluted 200 times, and their spectra were collected over the range of 250–500 nm.

The sulfide removal rates$R_{{HS}^{-}}$(mmol min^-1^) and the coulombic efficiencies${CE}_{{HS}^{-}}$(%) were calculated based on the Eq-1 and Eq-2^[1]^ respectively:

$R_{{HS}^{-}}=\frac{n_{{HS}^{-},to}- n_{{HS}^{-},t}}{\Delta t}$ (1)

where $n_{{HS}^{-},to}$is the initial number of sulfide moles in the anolyte, $n_{{HS}^{-},t}$ is the number of sulfide moles at each sampling time t and$\Delta t$ is the time of operation (min).

${CE}_{{HS}^{-}}=\frac{Z\times\left( n_{{HS}^{-},to}- n_{{HS}^{-},t} \right)\times F}{Q}\times100\%$ (2)

where z = 2 is the number of electrons (e^−^) involved in the reaction, F the Faraday constant (96485 C mol^-1^) and *Q* (C) is the total quantity of electric charge. In highly alkaline conditions (pH > 12), the coulombic efficiencies is calculated based on the direct oxidation of HS⁻ to S^0^, a 2-electron process.

**Calculation of HER Faradic efficiency (FE_H2_)**

Calculation of the Faradaic efficiency: The volume of hydrogen (*V_H2_*) was determined by drainage method, and the molar volume (*Vm*, at 25 °C and 1 atm) of gas was 24.5 L mol^-1^. The FE of H_2_ can be calculated as follow:

$n_{H_{2}}=\frac{V_{H2}}{V_{m}}\times100\%$ (3)

${FE}_{H_{2}}=\frac{V_{H2}}{V_{m}}\times100\%=\frac{n_{H2}\times N\times F}{j\times t}\times100\%$ (4)

where *Q*_H2_ denotes the quantity of electric charge needed to generate a specific product, *Q*_total_ denotes the total quantity of electric charge consumed, *n*_H2_ denotes the molar mass that generates a specific product.

**Calculation details in saving of energy consumption**

When hydrogen was produced with the traditional HER + OER system, the energy consumption could be calculated by the formula: W = *U* × *I* × *t* = *U* × *Q*, where *U* is the operating voltage of the two-electrode systems (V), *I* is the corresponding current (A), *t* is the electrolysis time (s), and *Q* is the passed charge through the electrode (C). The electrical energy consumption of the HER +OER system was depicted as W1 = *U*1 × *Q*1, and that of the HER + SOR system was W2 = *U*2 × *Q*2. When a certain amount of hydrogen was generated, a corresponding quantity of electrons was required (*Qhydrogen*), so the total quantity of electrons passed through electrodes can be represented as *Q* = *Q_hydrogen_/FE*. Thus, when the same amount of hydrogen was produced, the electrical energy saving of the HER + SOR system compared to the HER + OER system can be calculated as the following equations:^[2]^

$$\eta=\frac{W1-W2}{W1}\times100\%=\frac{Q1\times U1-Q2\times U2}{Q1\times U1}\times100\%=\frac{\frac{Q_{hydrogen}}{FE1}\times U1-\frac{Q_{hydrogen}}{FE2}\times U2}{\frac{Q_{hydrogen}}{FE1}\times U1}\times100\%=\left[ 1-\frac{U2\times FE1}{U1\times FE2} \right]\times100\%$$

Assuming that all the electrons were contributed to hydrogen production, which means FE values were at 100%, according to the equations, the energy consumption saving as the OER was replaced by SOR can be calculated as follows:

The required cell voltage of HER + OER system was 1.42 and 1.53 V at 100 and 300 mA cm^-2^ while those of the HER + SOR system were 0.88 and 0.99 V. The energy consumption savings at 100 and 300 mA cm^-2^ were calculated as follows:

$$\eta_{100}=\left( 1-\frac{U2}{U1} \right)\times100\%=\left( 1-\frac{0.88}{1.42} \right)\times100\%=38.0\%$$

$$\eta_{300}=\left( 1-\frac{U2}{U1} \right)\times100\%=\left( 1-\frac{0.99}{1.53} \right)\times100\%=35.2\%$$

**2.** **Supporting Figures and Tables**


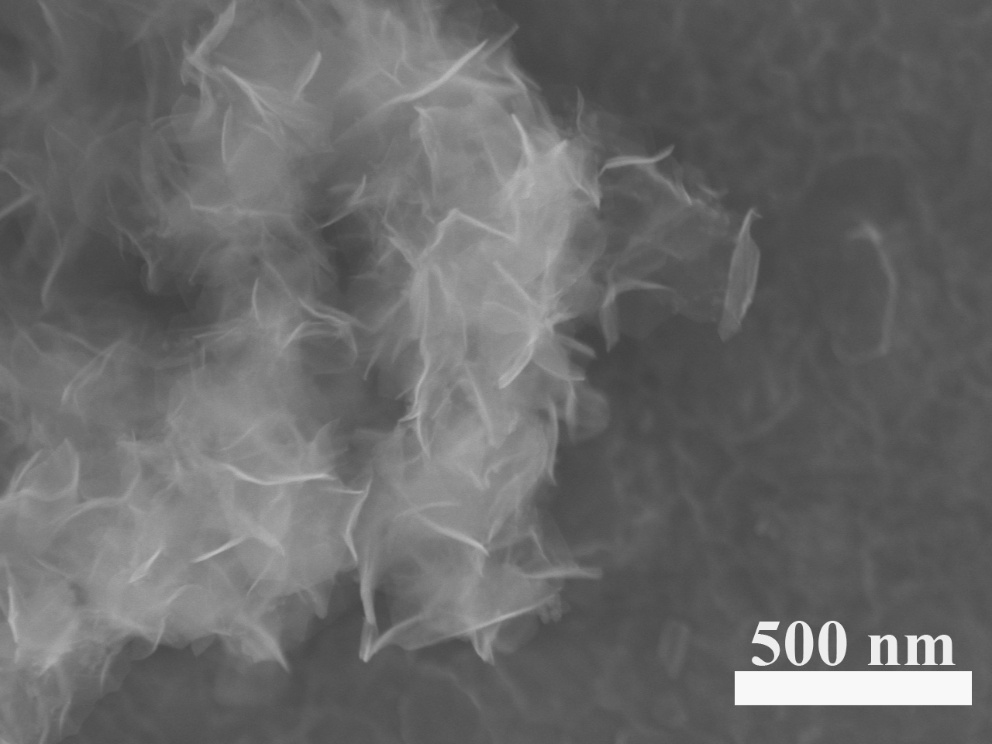


**Figure S1**. SEM image of Sc-NiFe-LDH.


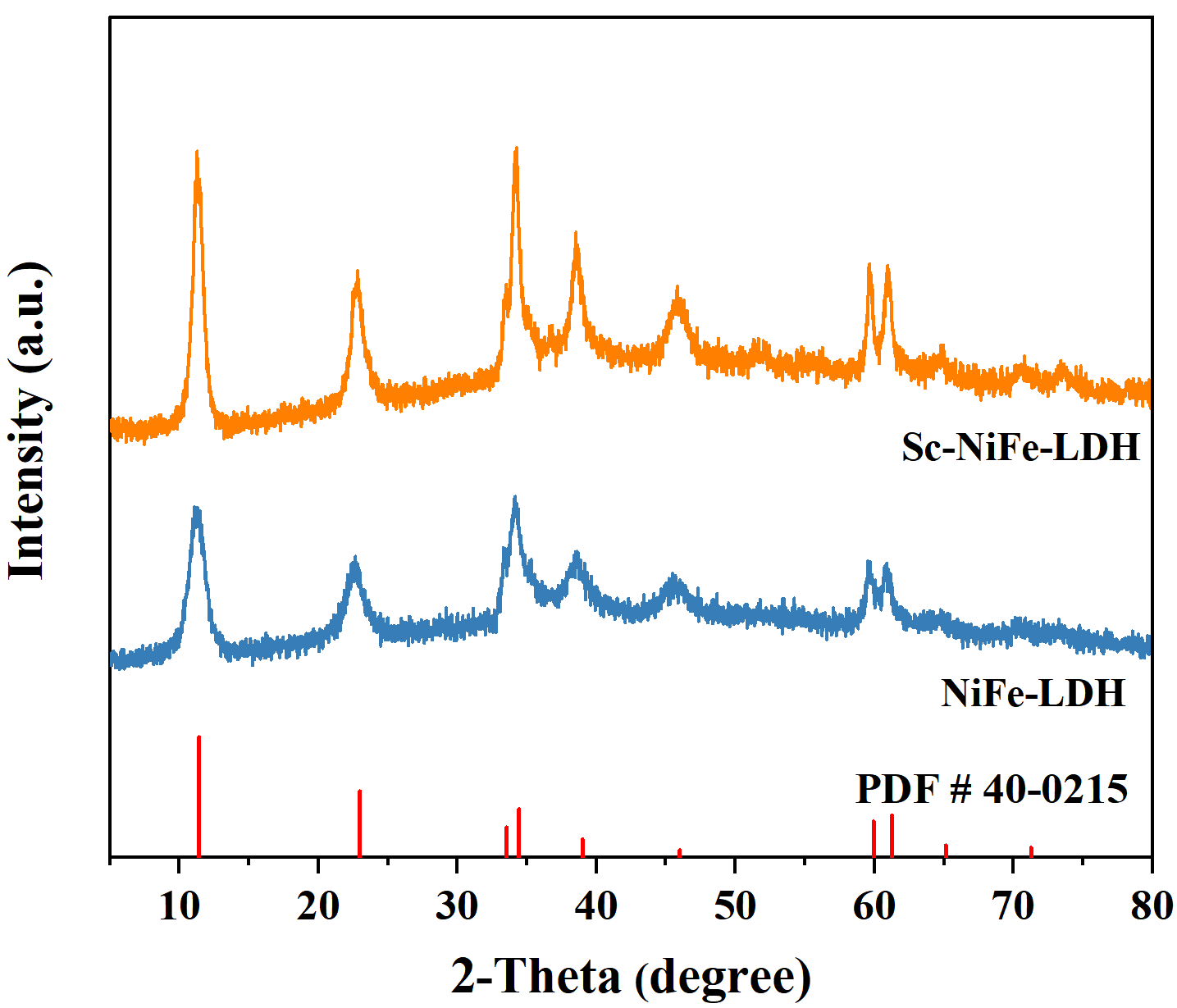


**Figure S2.** XRD pattern of NiFe-LDH and Sc-NiFe-LDH.^[3]^ Copyright 2024 Royal Society of Chemistry


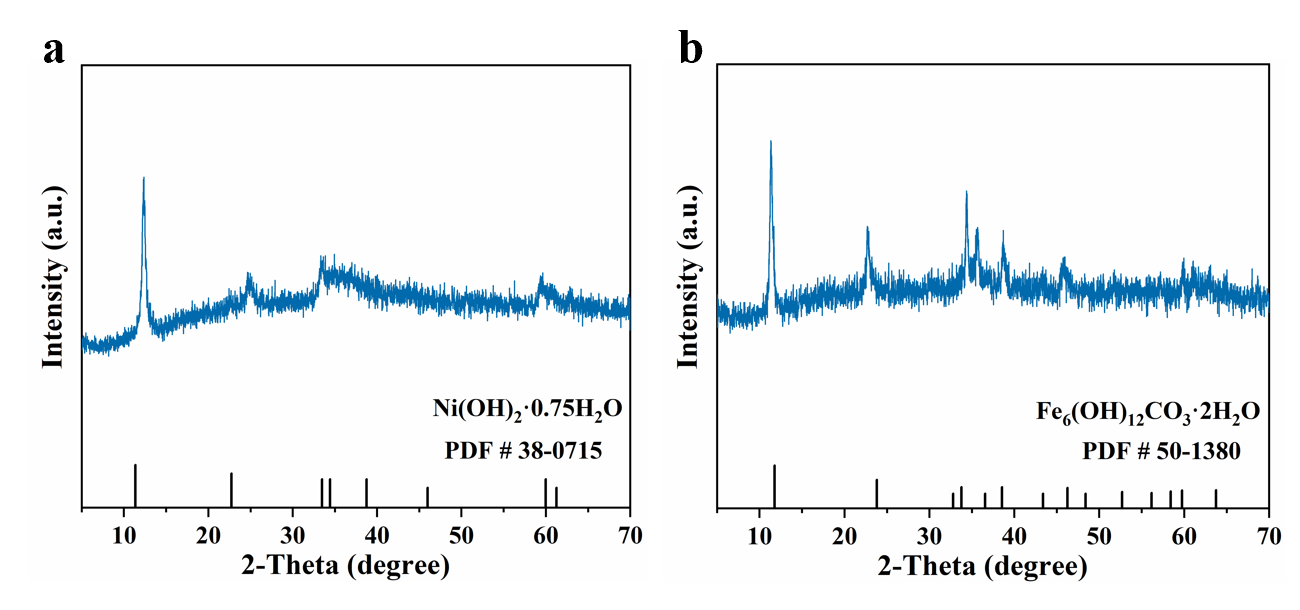


**Figure S3.** XRD pattern of Ni(OH)_2_ and Fe_6_(OH)_12_CO_3_·2H_2_O.


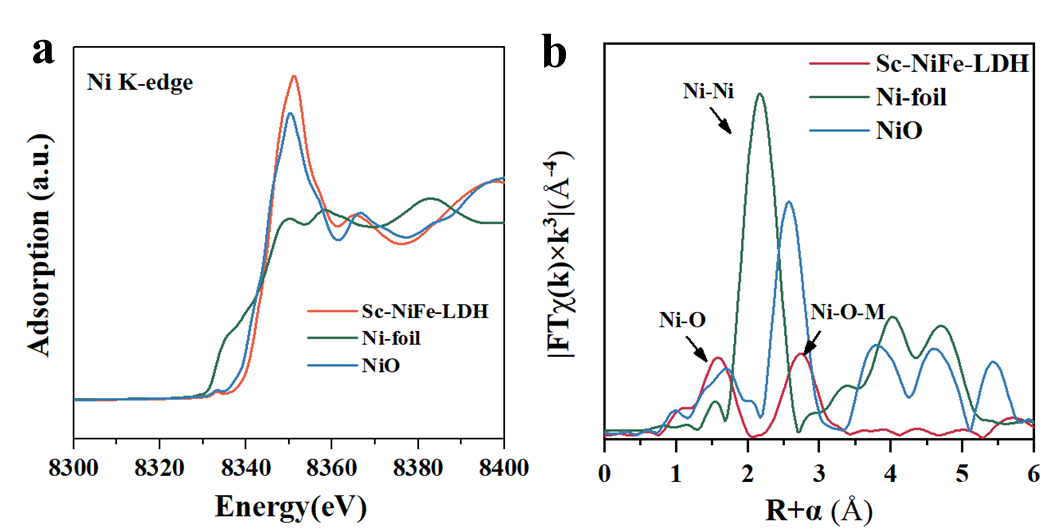


**Figure S4.** (a) Ni K-edge XANES spectra with magnified marked region inserted, (b) Fourier-transform spectra from EXAFS at the Ni K-edge.


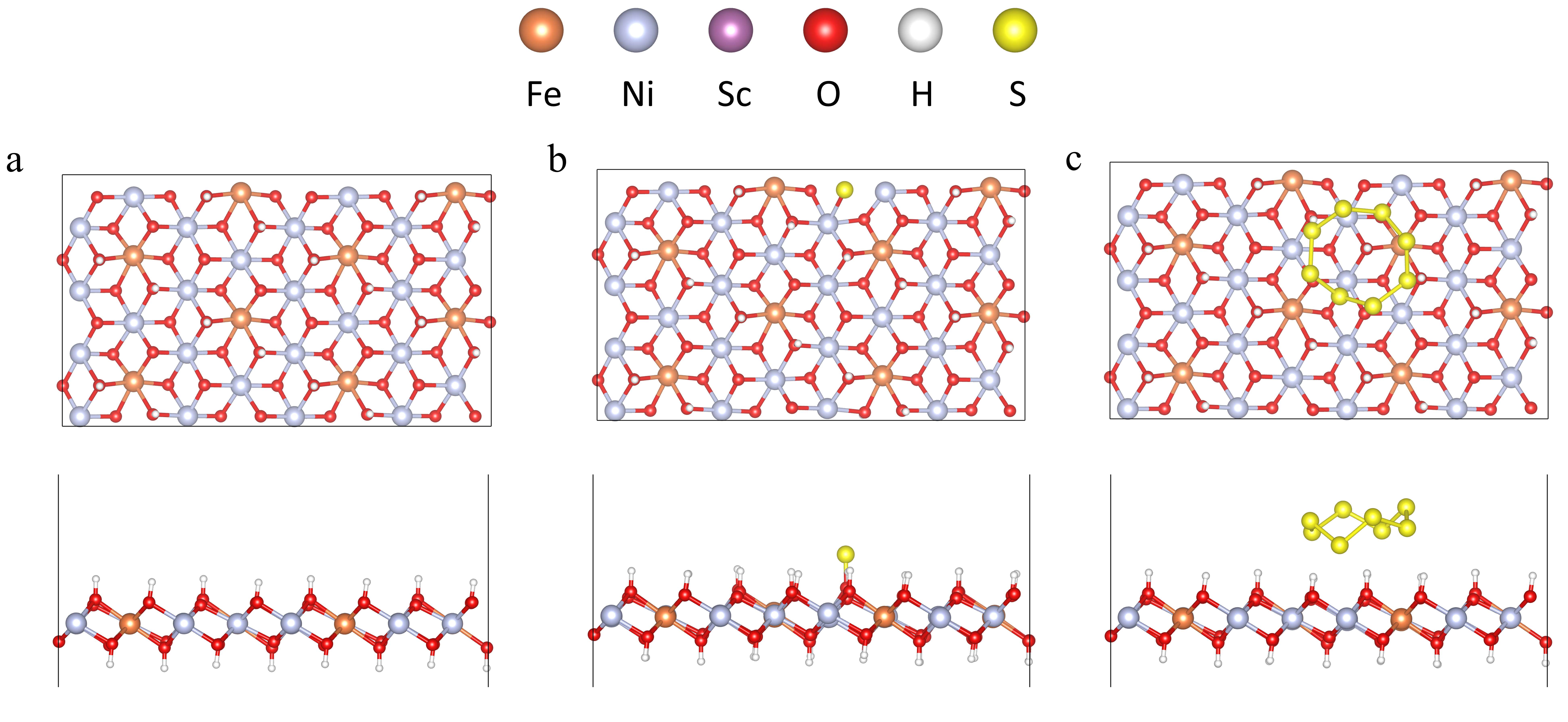


**Figure S5.** Top and side views of the NiFeOOH(001) surface: (a) optimized structure, (b) structure with adsorbed sulfur atom (*S), and (c) structure with adsorbed elemental sulfur (*S₈).


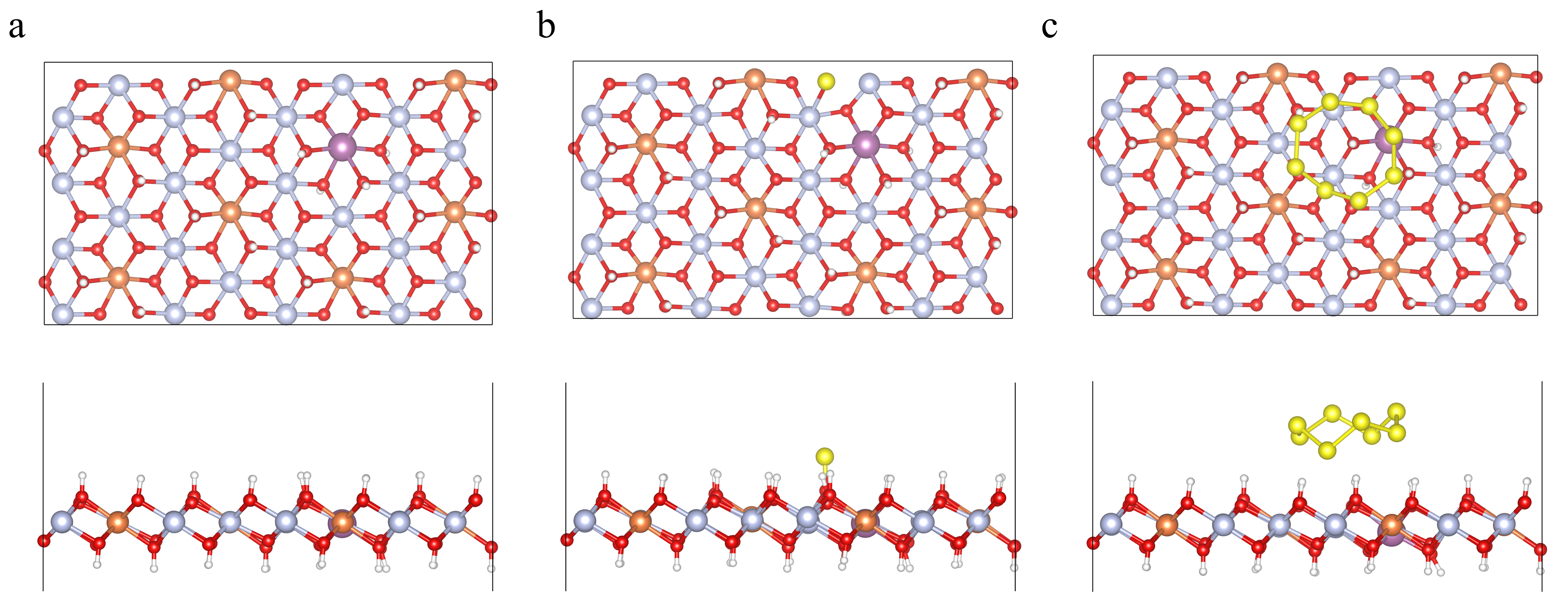


**Figure S6.** Top and side views of the NiFeOOH(001)-Sc surface: (a) optimized structure, (b) structure with adsorbed sulfur atom (*S), and (c) structure with adsorbed elemental sulfur (*S₈).


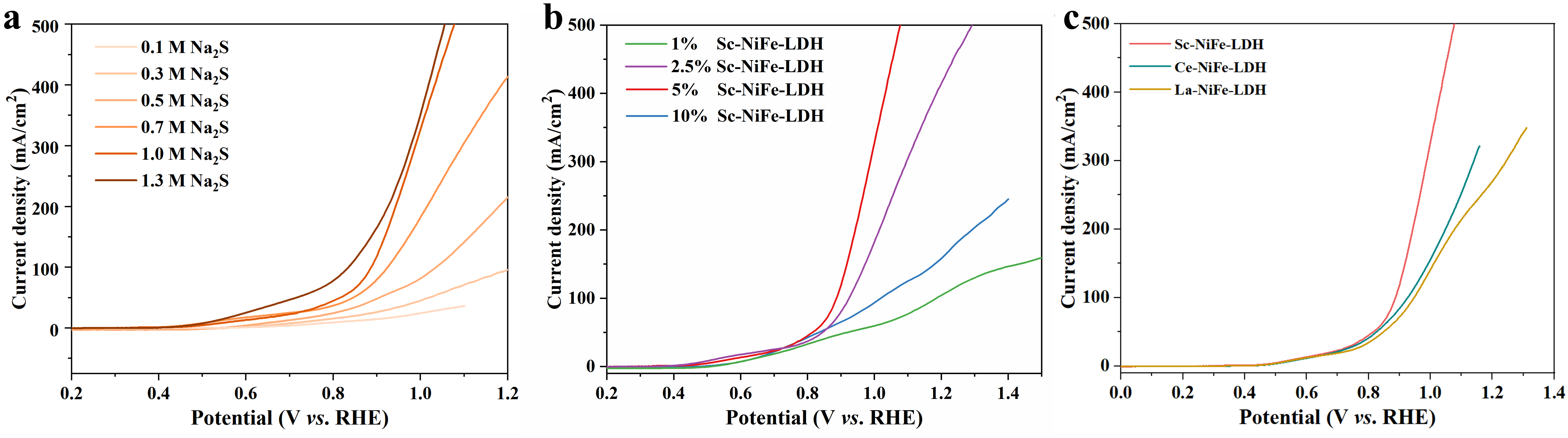


**Figure S7**. (a) LSV curves of Sc-NiFe-LDH for SOR in different concentrations of Na_2_S; (b) LSV of Sc-NiFe-LDH with different Sc contents in 1 m NaOH with 1 m Na_2_S; (c) LSV of Sc-NiFe-LDH, Ce-NiFe-LDH, La-NiFe-LDH.


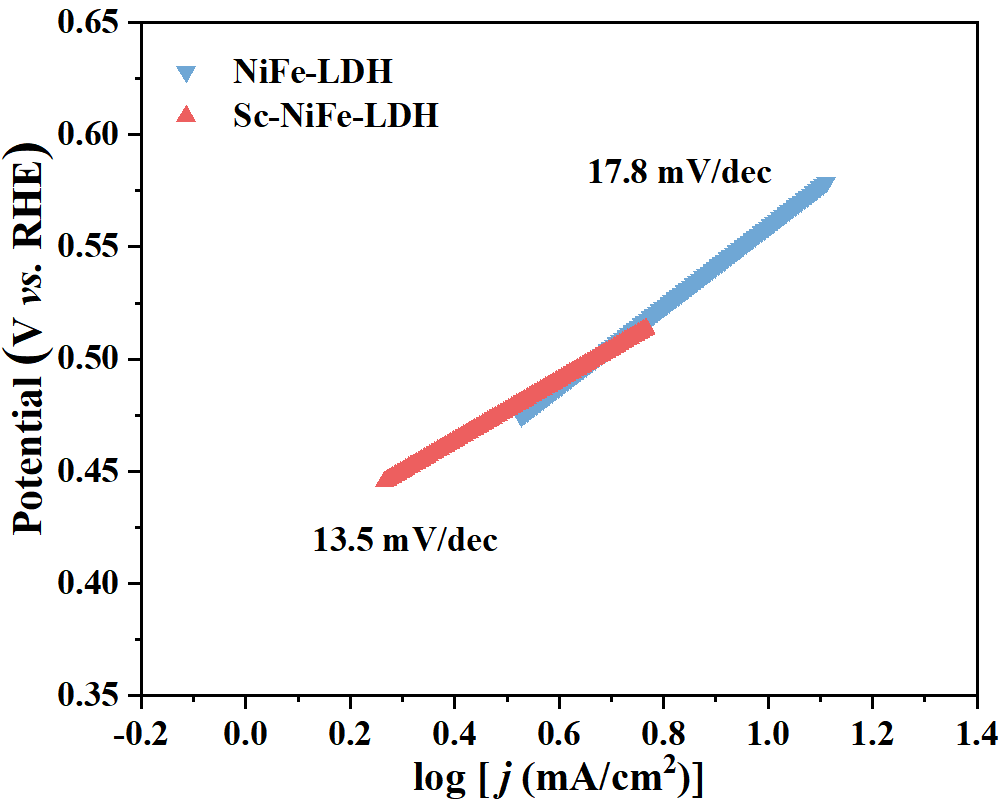


**Figure S8.** Tafel plots of Sc-NiFe-LDH and NiFe-LDH.





**Figure S9**. EIS plots of Sc-NiFe-LDH and NiFe-LDH for SOR in 1 m Na_2_S + 1 m NaOH electrolyte.


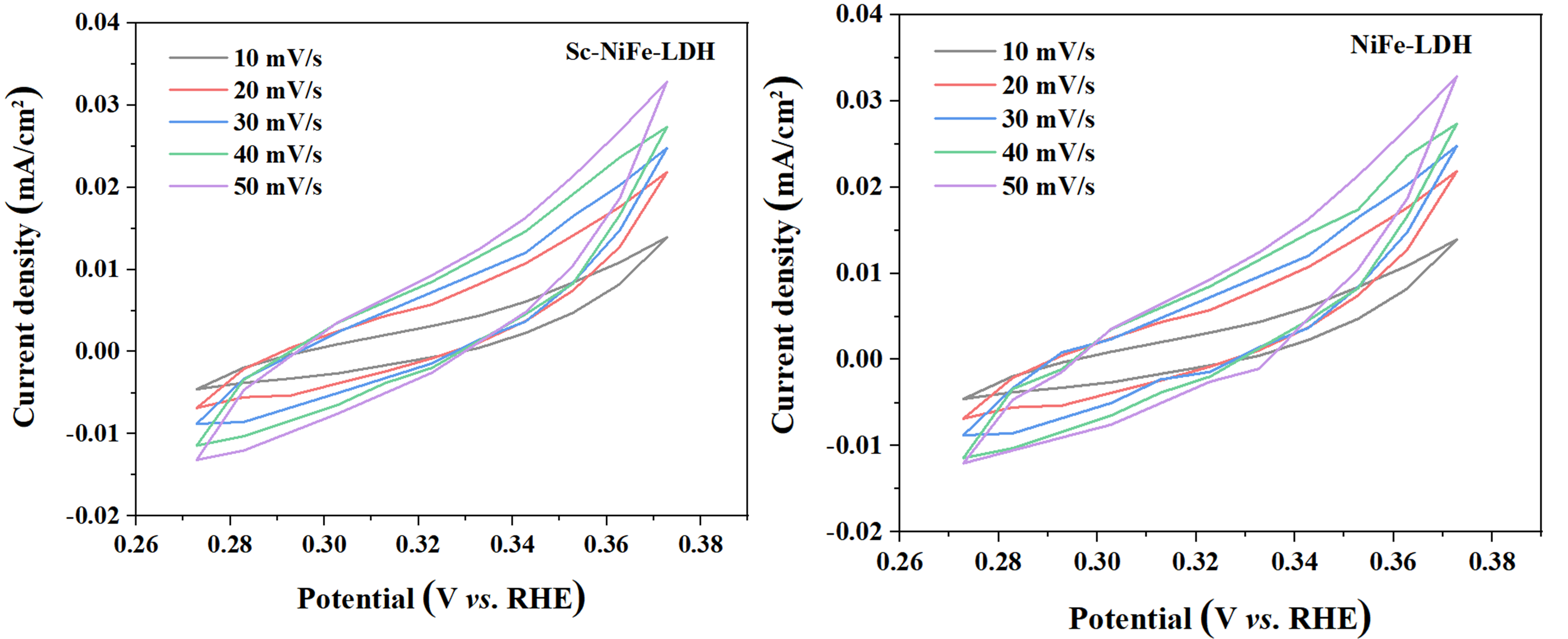


**Figure S10**. The CV curves under different scan rates in the non-Faraday region of

(a) Sc-NiFe-LDH and (b) NiFe-LDH.


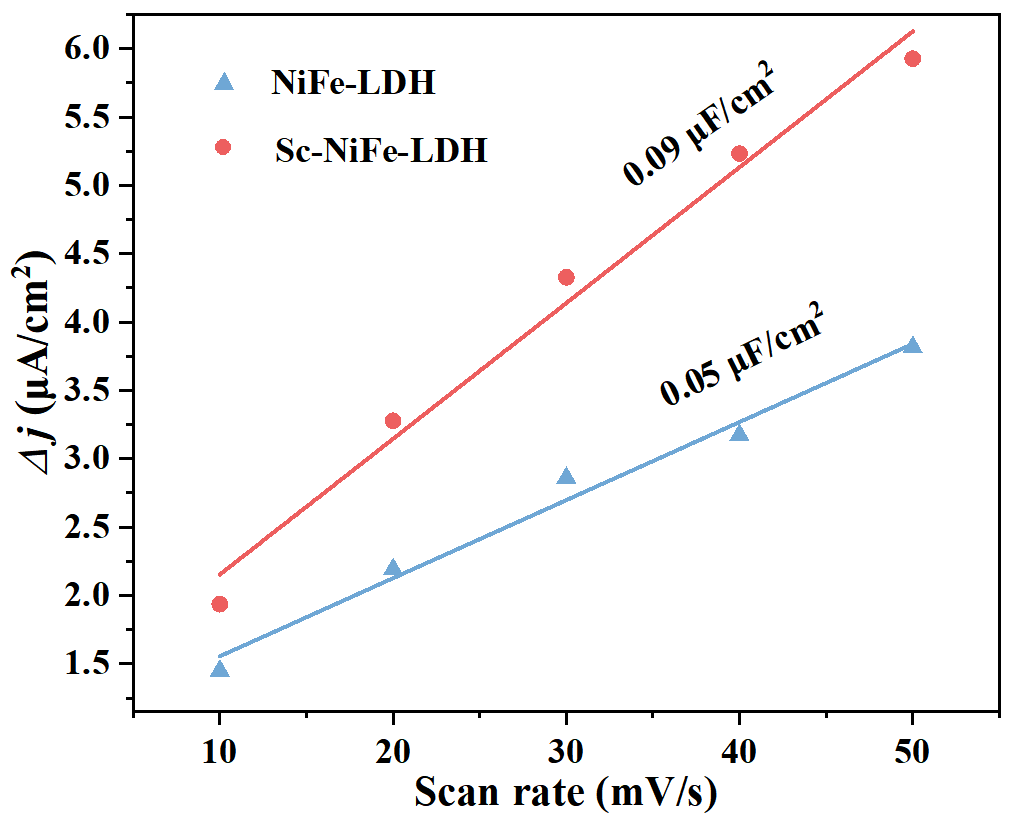


**Figure S11**. C_dl_ of catalysts derived from the current density versus the scan rate.


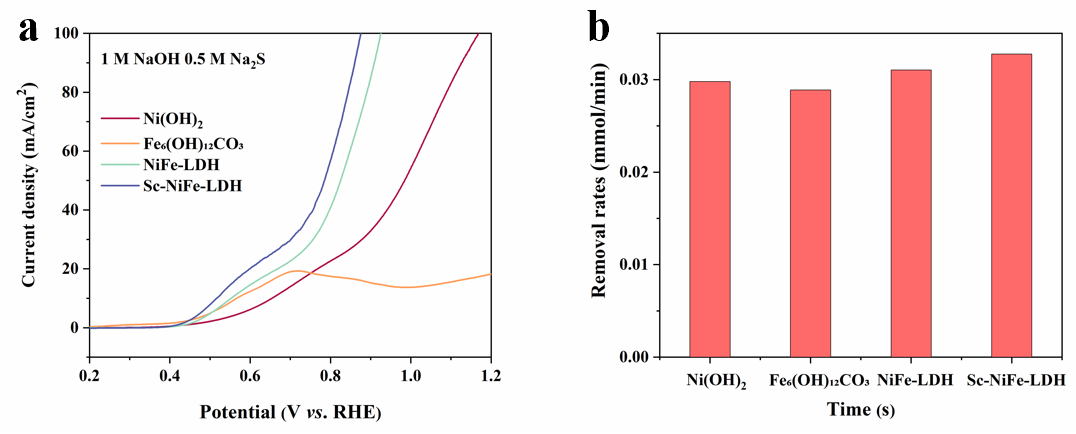


**Figure S12**. (a) LSV curves and (b) removal rates of Ni(OH)_2_, Fe_6_(OH)_12_CO_3_, NiFe-LDH and Sc-NiFe-LDH for SOR in 1 m NaOH with 0.5 M Na_2_S.


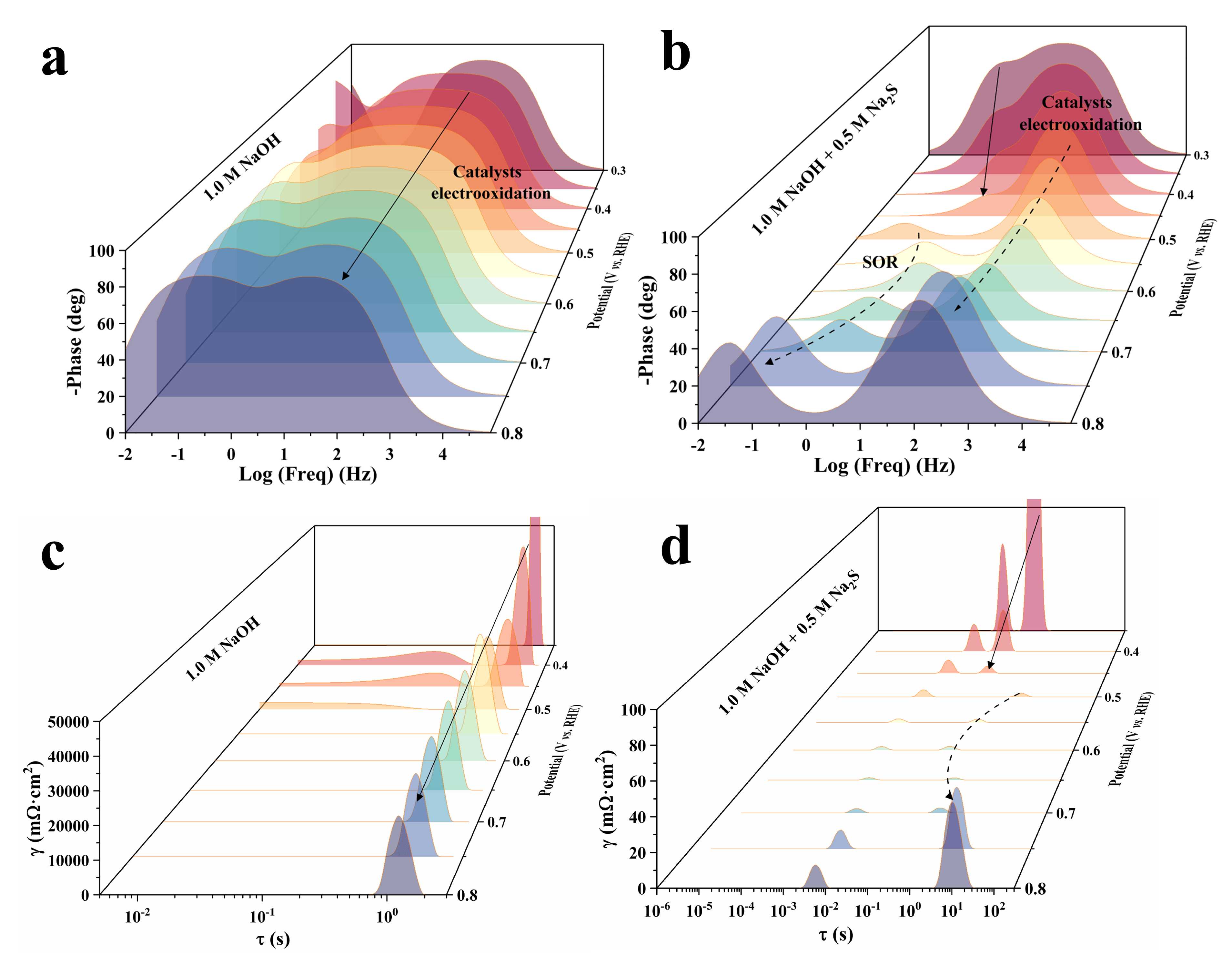


**Figure S13**. Bode phase plots and corresponding DRT analysis derived from in situ EIS measurements of Sc-NiFe-LDH in 1.0 M NaOH ((a), (c)) and 1.0 M NaOH + 0.5 M Na₂S ((b), (d)).


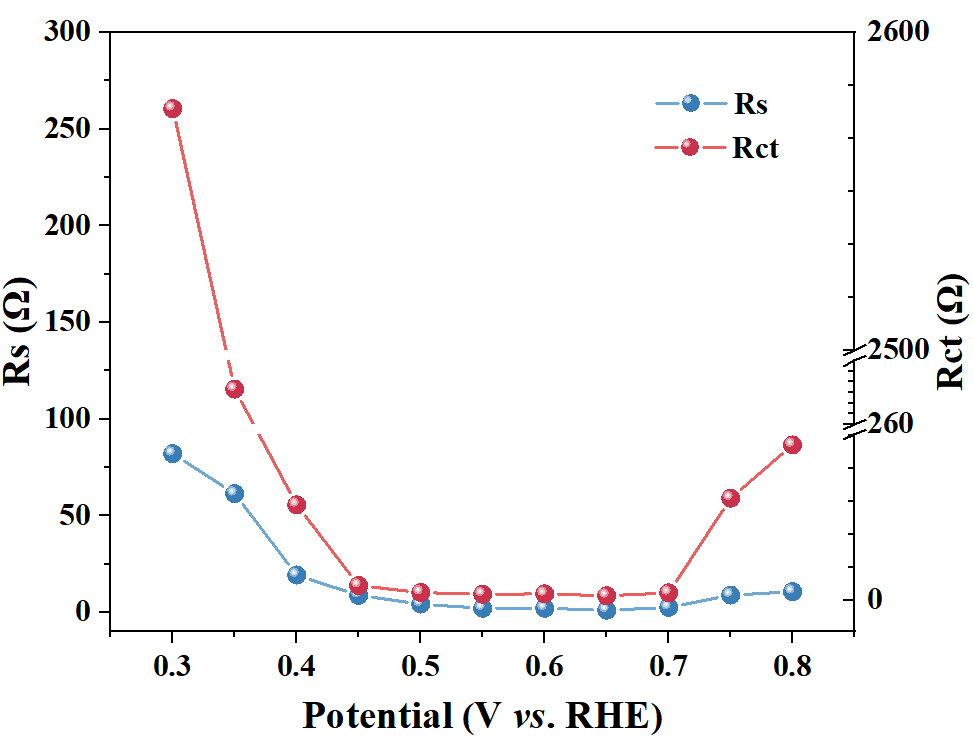


**Figure S14**. Charge-transfer resistance (Rct) and solution resistance (Rs) at various potentials.





**Figure S15**. XRD pattern of S_8_ (powder collected from the electrode surface).


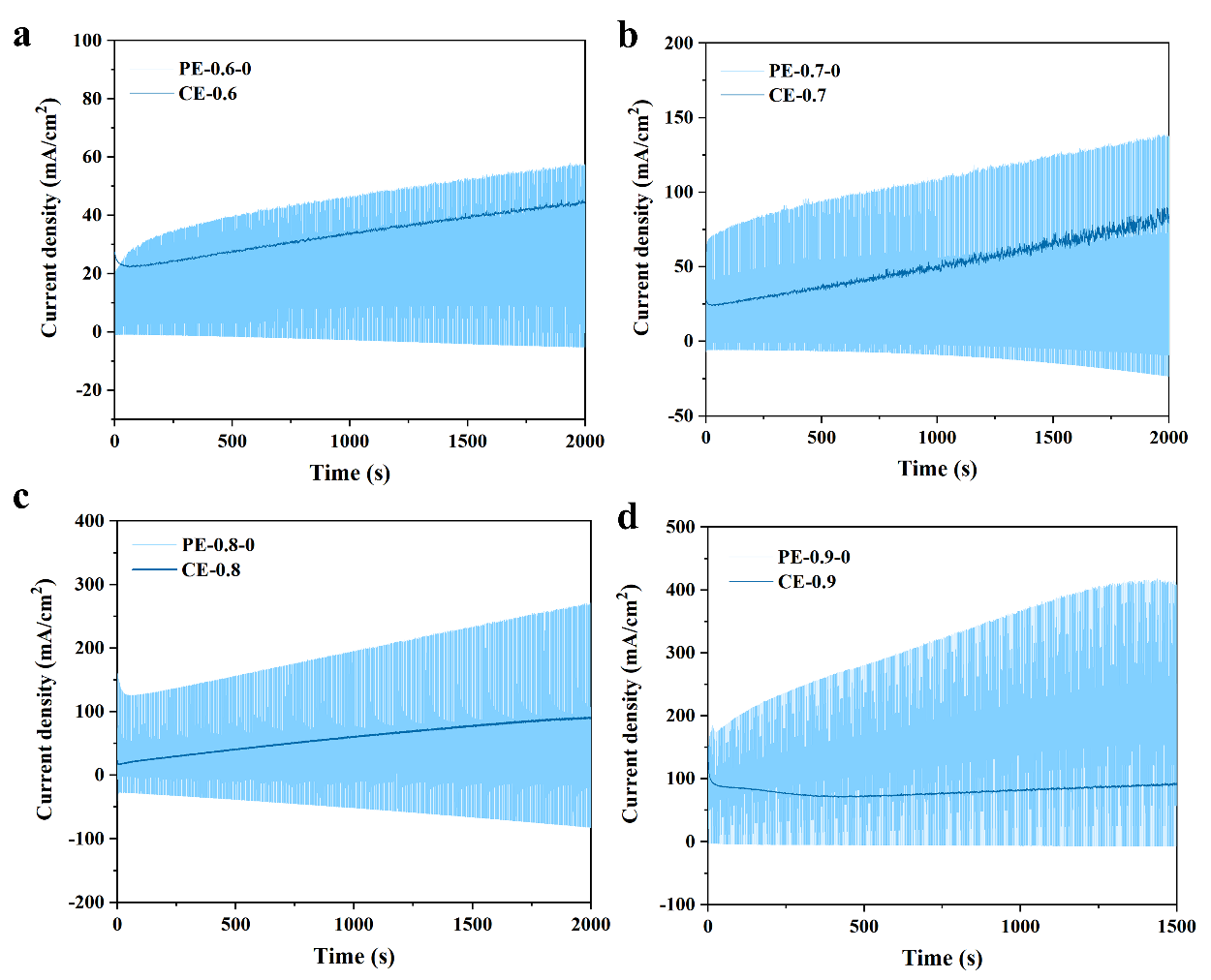


**Figure S16**. Chronoamperometric curves recorded in CE and PE modes at different potentials: (a) 0.6 V_RHE_, (b) 0.7 V_RHE_ (c) 0.8 V_RHE_, and (d) 0.9 V_RHE._ For PE mode, pulses of 1 s were applied at alternating high and low potentials.


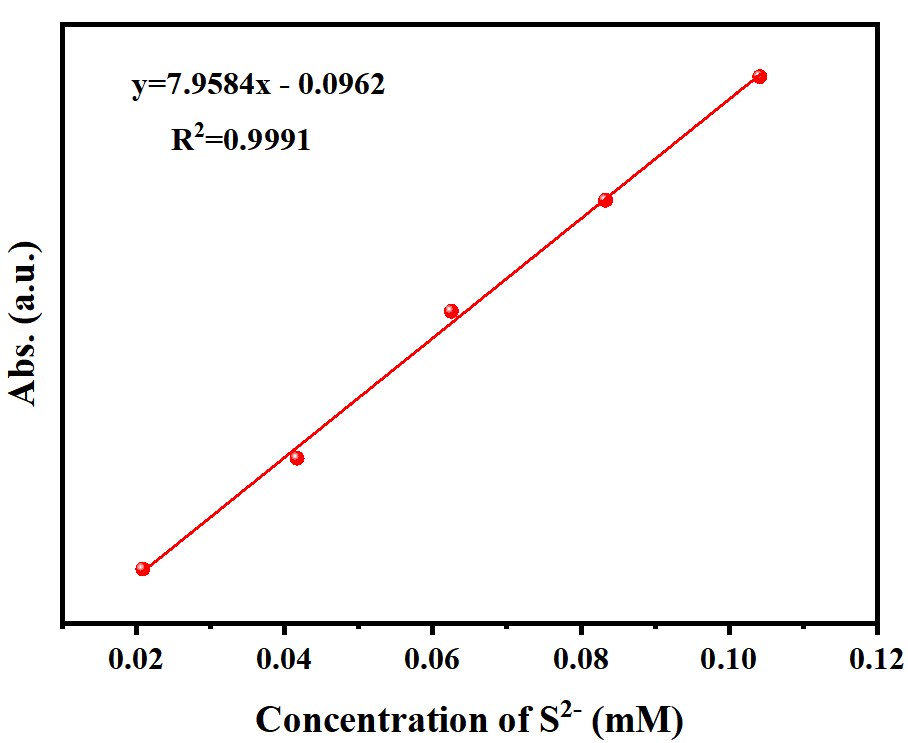


**Figure S17**. Standard curve of concentration for S^2−^ .


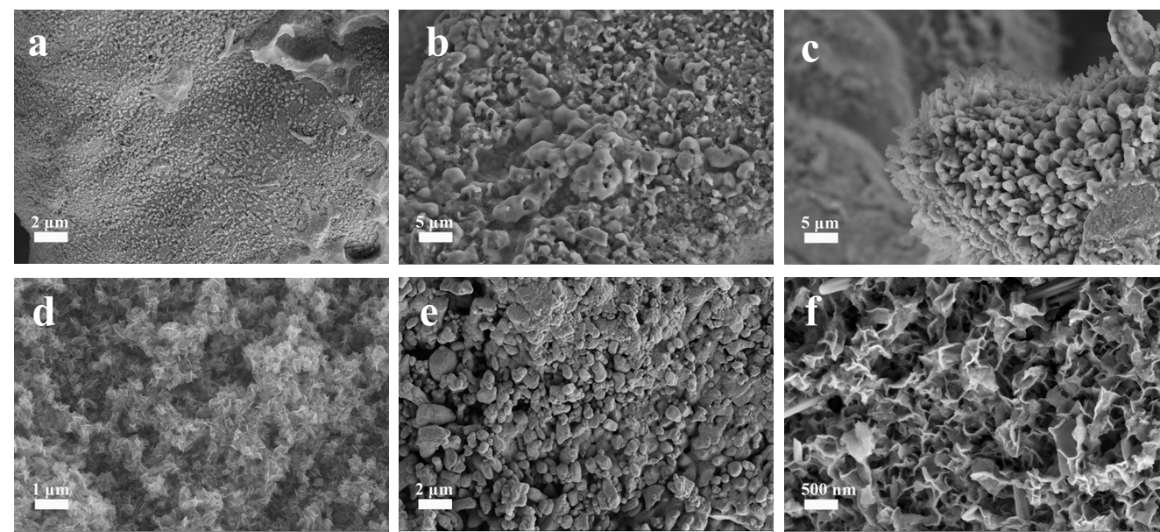


**Figure S18.** SEM images of Sc-NiFe-LDH under different electrochemical conditions: (a–c) after CE at 0.7 V, 0.8 V, and 0.9 V vs. RHE, respectively; (d) as-prepared Sc-NiFe-LDH; (e) after constant current electrolysis at 100 mA cm^-2^; (f) after PE at 0.9 V for 2 s and 0 V for 1 s.


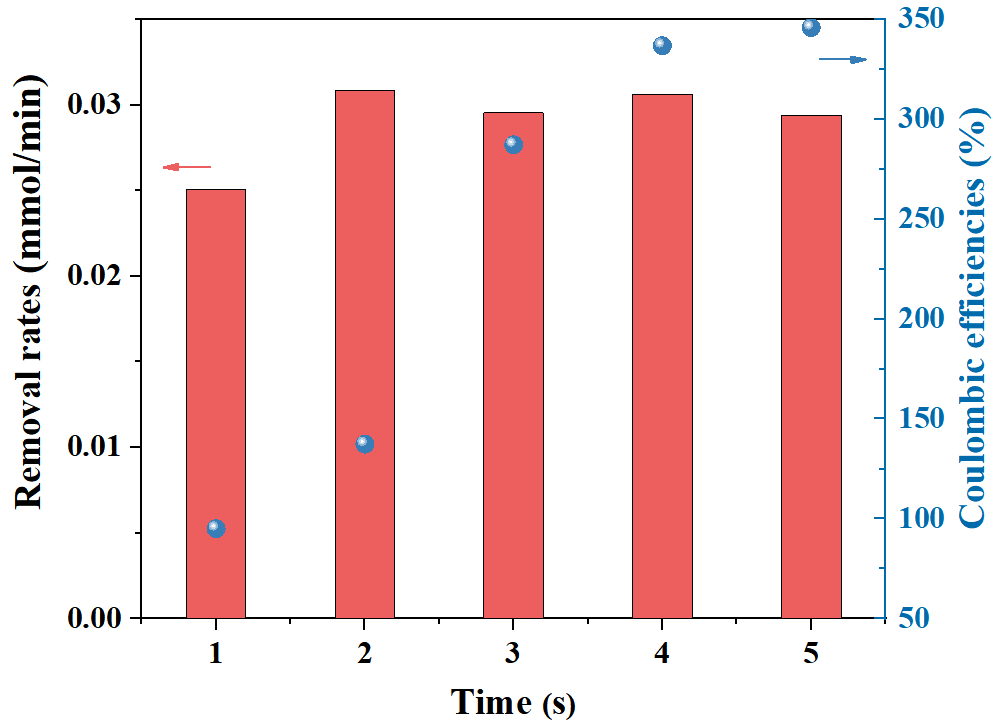


**Figure S19**. Removal rates and Coulombic efficiencies of concentration for S^2−^.


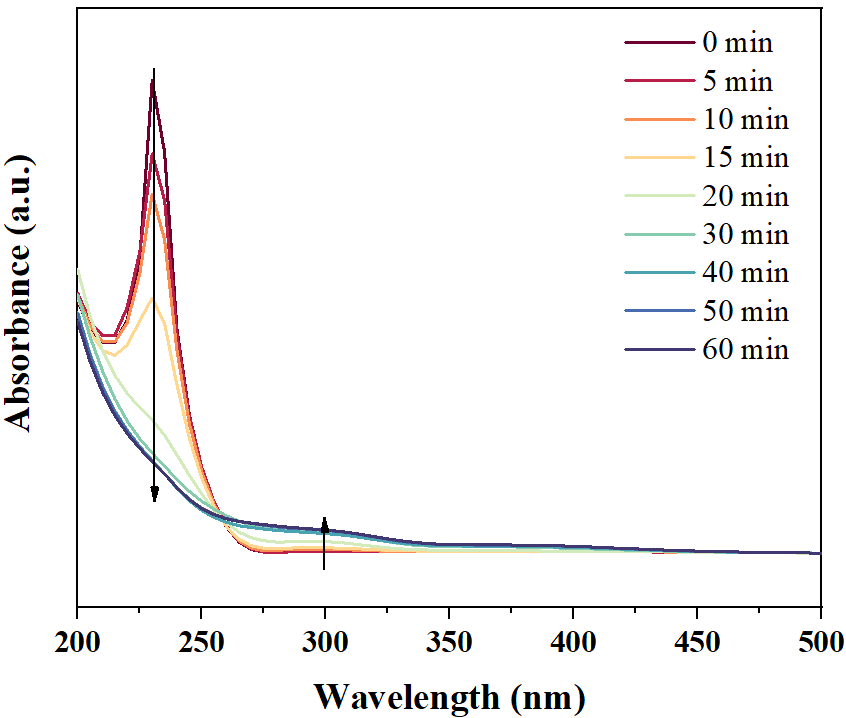


**Figure S20**. UV-vis spectra of anolyte during SOR at PE mode (E_0.9_ = 2 s, E_0_ = 1 s).


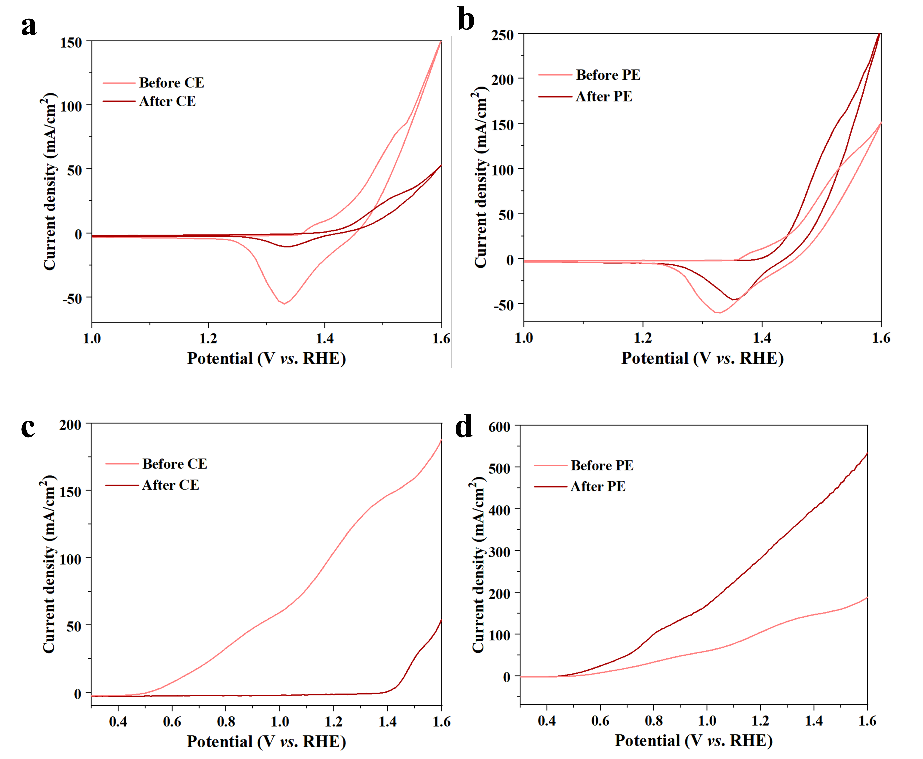


**Figure S21.** CV and LSV curves before and after the I–t test in CE (0.9 V_RHE_) and PE (0.9 V_RHE_ for 1 s; 0 V for 1 s) modes: (a, b) CV curves; (c, d) LSV curves.


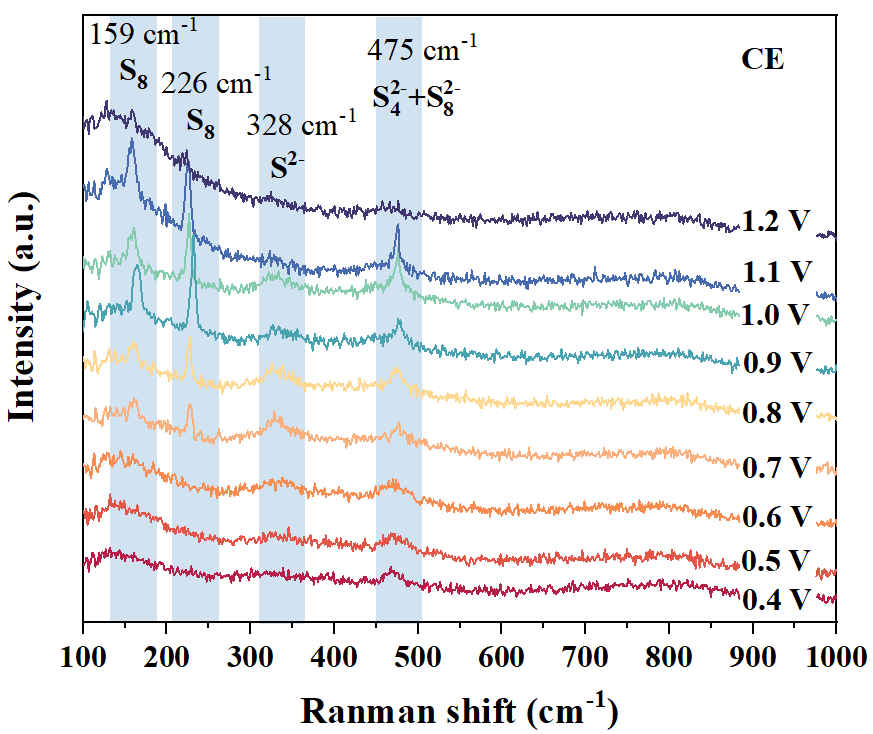


**Figure S22.** In situ Raman spectra of Sc-NiFe-LDH catalysts in 0.5 M Na₂S + 1 m NaOH at various applied potentials.

The decrease in Raman peak intensity at 1.2 V mainly originates from the *in situ* oxidation and desorption of surface-adsorbed sulfur intermediates (e.g., S* and polysulfides) into soluble oxidized sulfur species. This reduces the surface coverage of Raman-active species and consequently weakens the Raman signal. In addition, the intensity variation also be partially influenced by potential-dependent Raman enhancement effects, as changes in the electrode/electrolyte interfacial structure and surface charge distribution can modulate the Raman scattering efficiency.


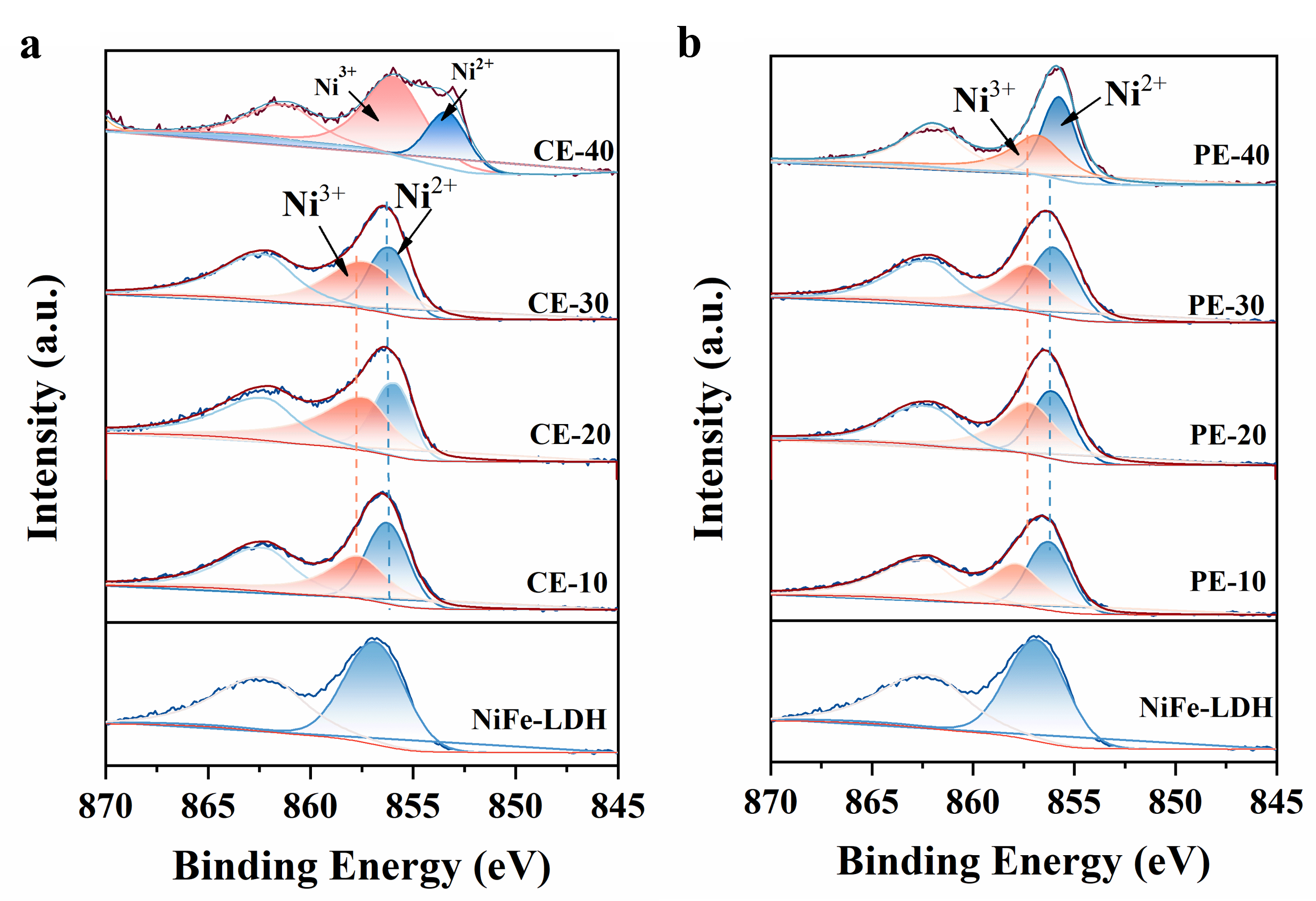


**Figure S23.** Ex situ Ni 2p_3/2_ spectra of Sc-NiFe-LDH electrodes collected after 10, 20, 30, and 40 min in 1 m NaOH containing 0.5 M Na₂S at (a) CE mode and (b) PE mode.


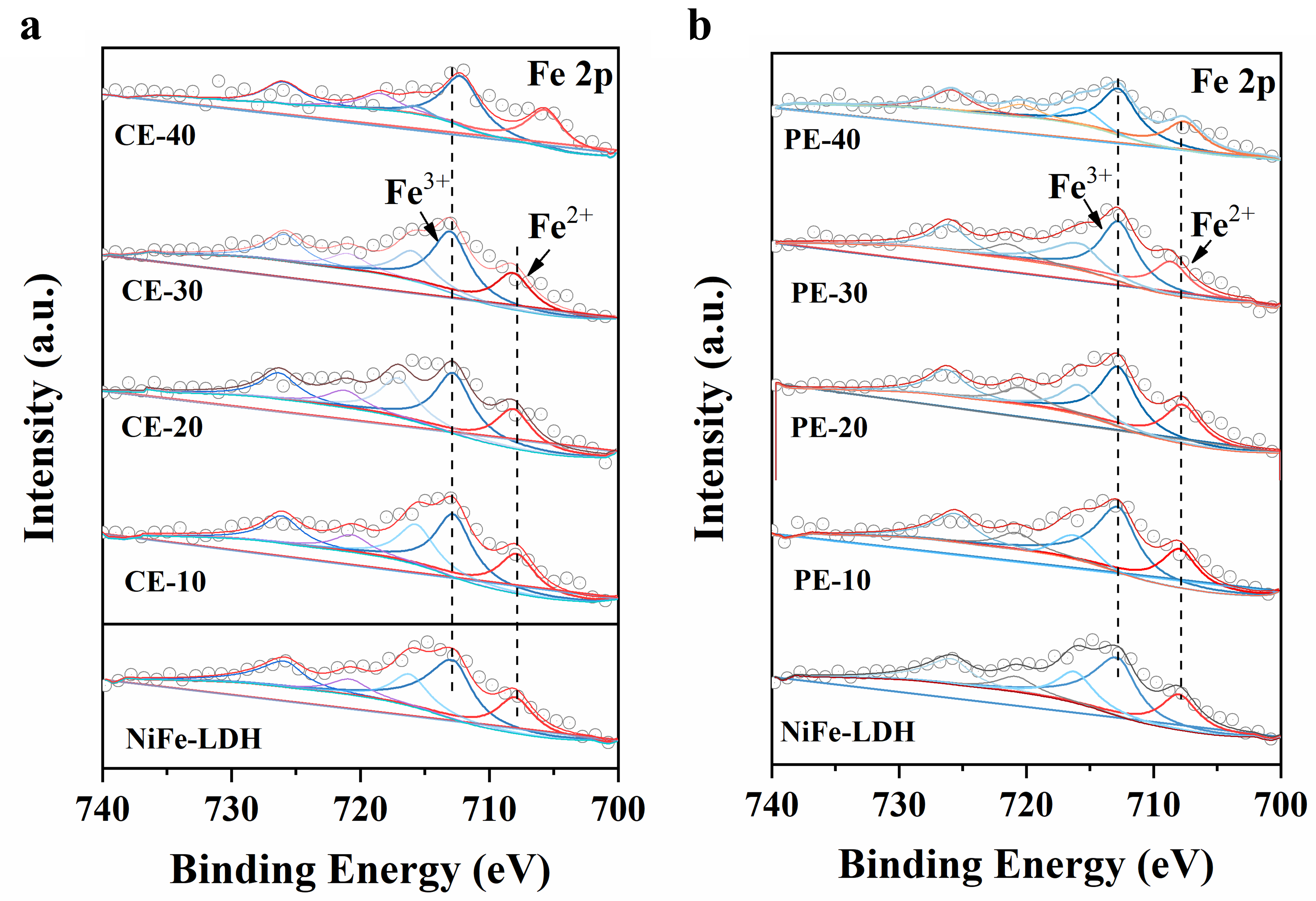


**Figure S24.** Ex situ Fe 2p spectra of Sc-NiFe-LDH electrodes collected after 10, 20, 30, and 40 min in 1 m NaOH containing 0.5 M Na₂S at (a) CE mode and (b) PE mode.


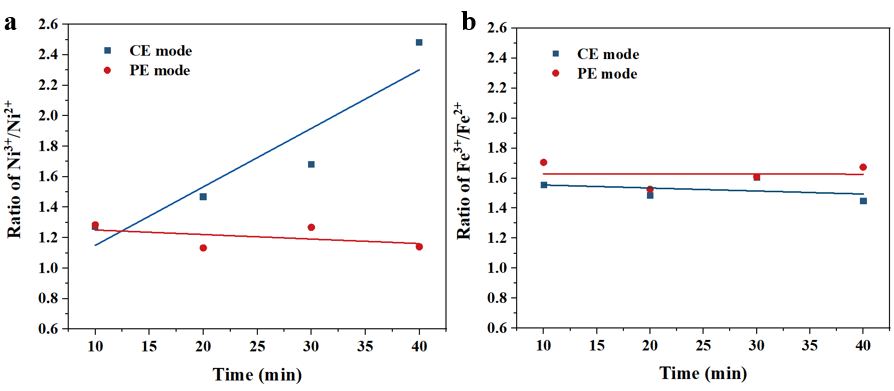


**Figure S25.** The change in the ratio of M^3+^/M^2+^ with time at CE and PE mode, (a) Ni^3+^/Ni^2+^; (b) Fe^3+^/Fe^2+^.

**
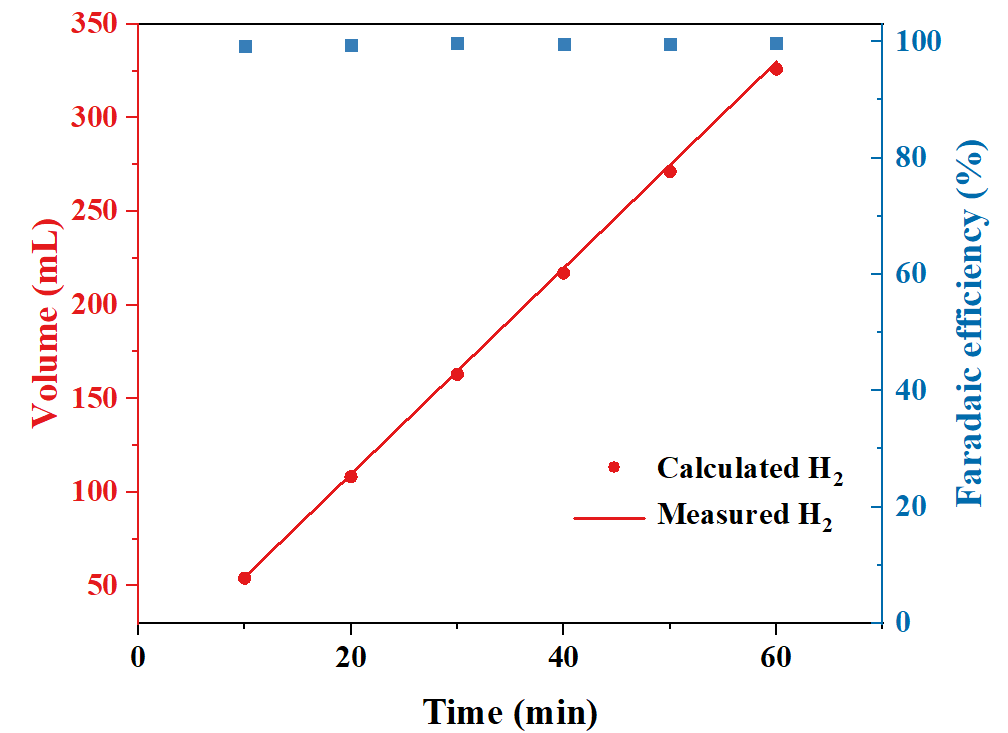
**

**Figure S26.** Evolution rates of H_2_ production in electrolysis process


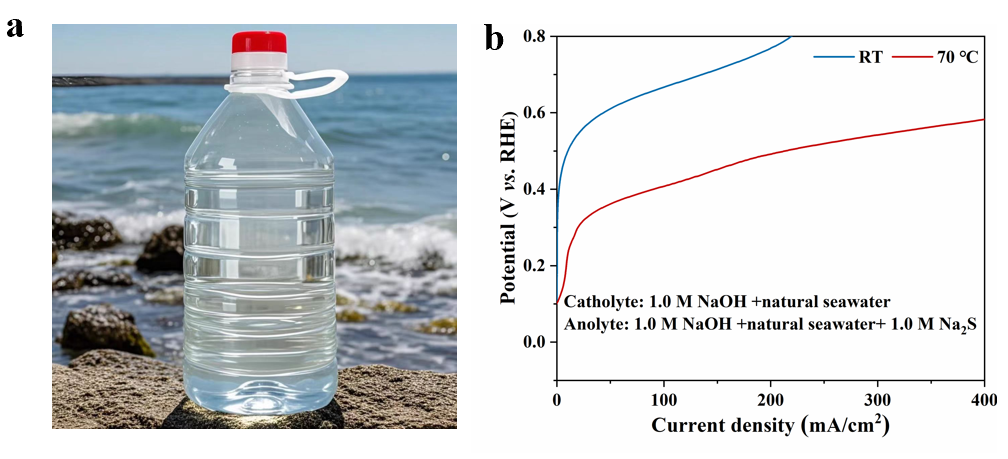


**Figure S27.** (a) The optical photograph of seawater collected from the Bohai Sea, China;(b) LSV curves at room temperature (RT) and 70 °C.


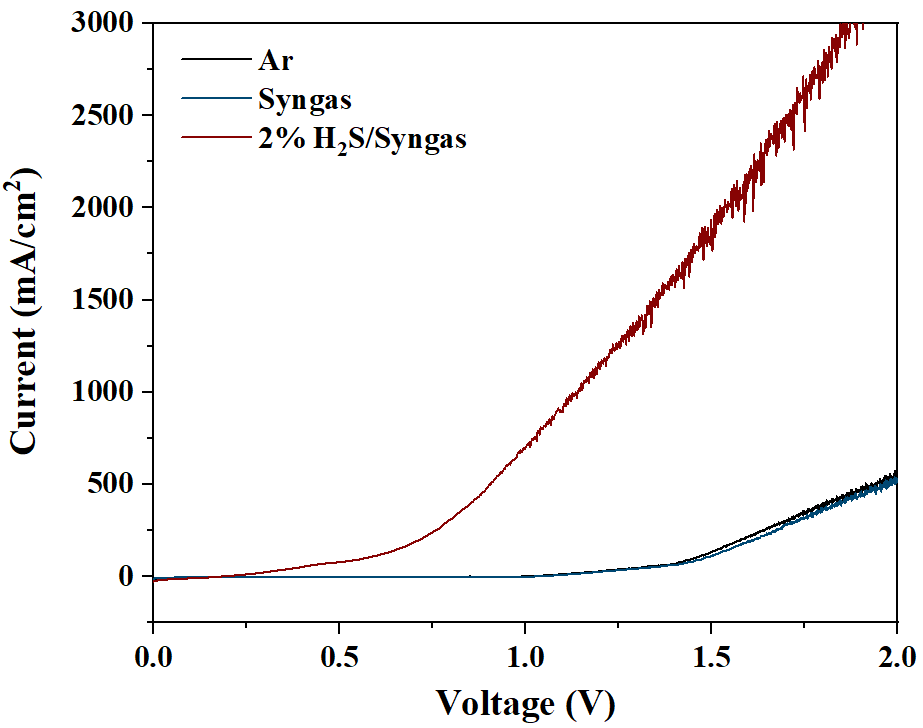


**Figure S28.** LSV curves of Sc-NiFe-LDH in different electrolytes.


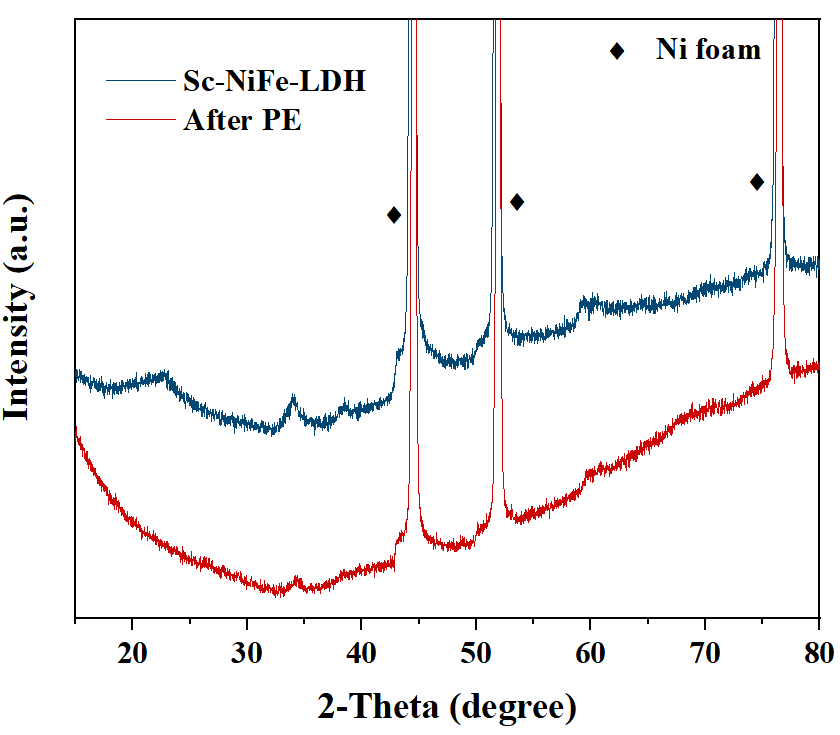


**Figure S29.** XRD patterns of Sc-NiFe-LDH before and after the PE stability test.


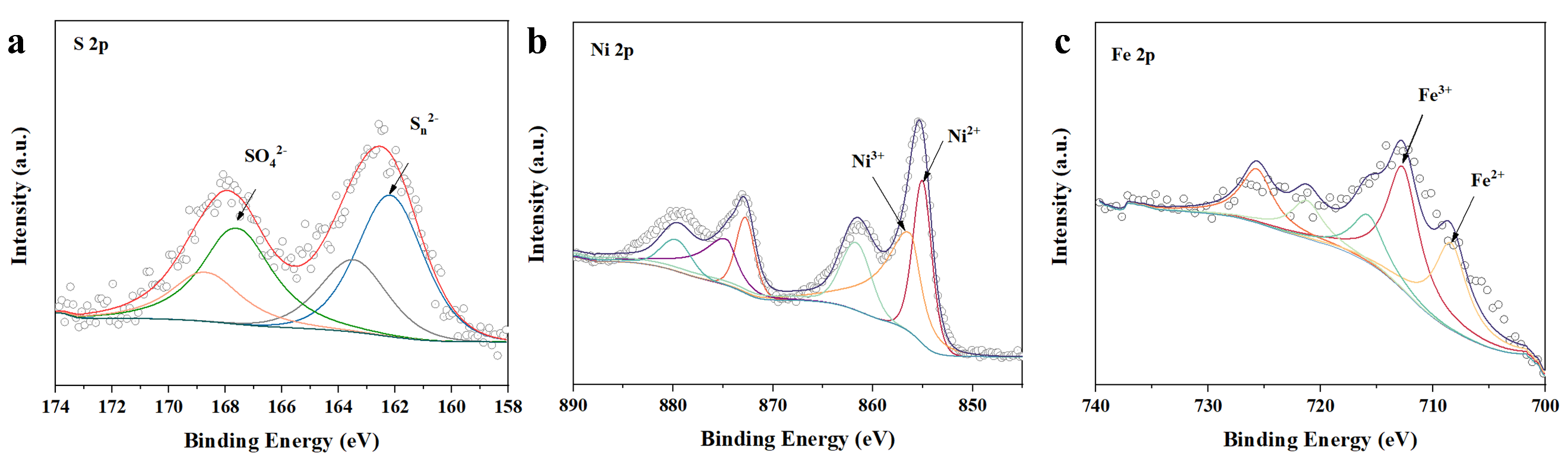


**Figure S30.** XPS spectra of Sc-NiFe-LDH after the PE stability test (a) S 2p, (b) Ni 2p, (c) Fe 2p.


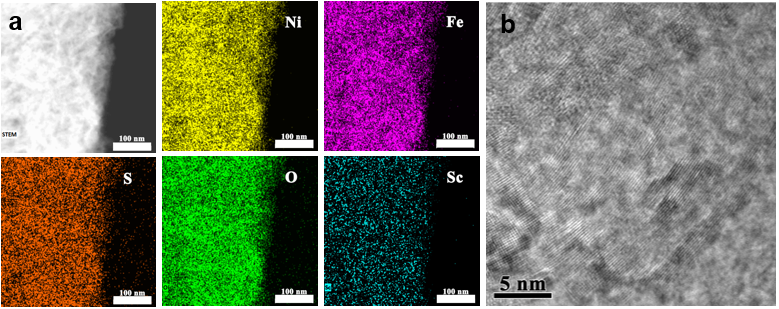


**Figure S31.** (a) Elemental mapping; (b) HRTEM after the CE stability test.


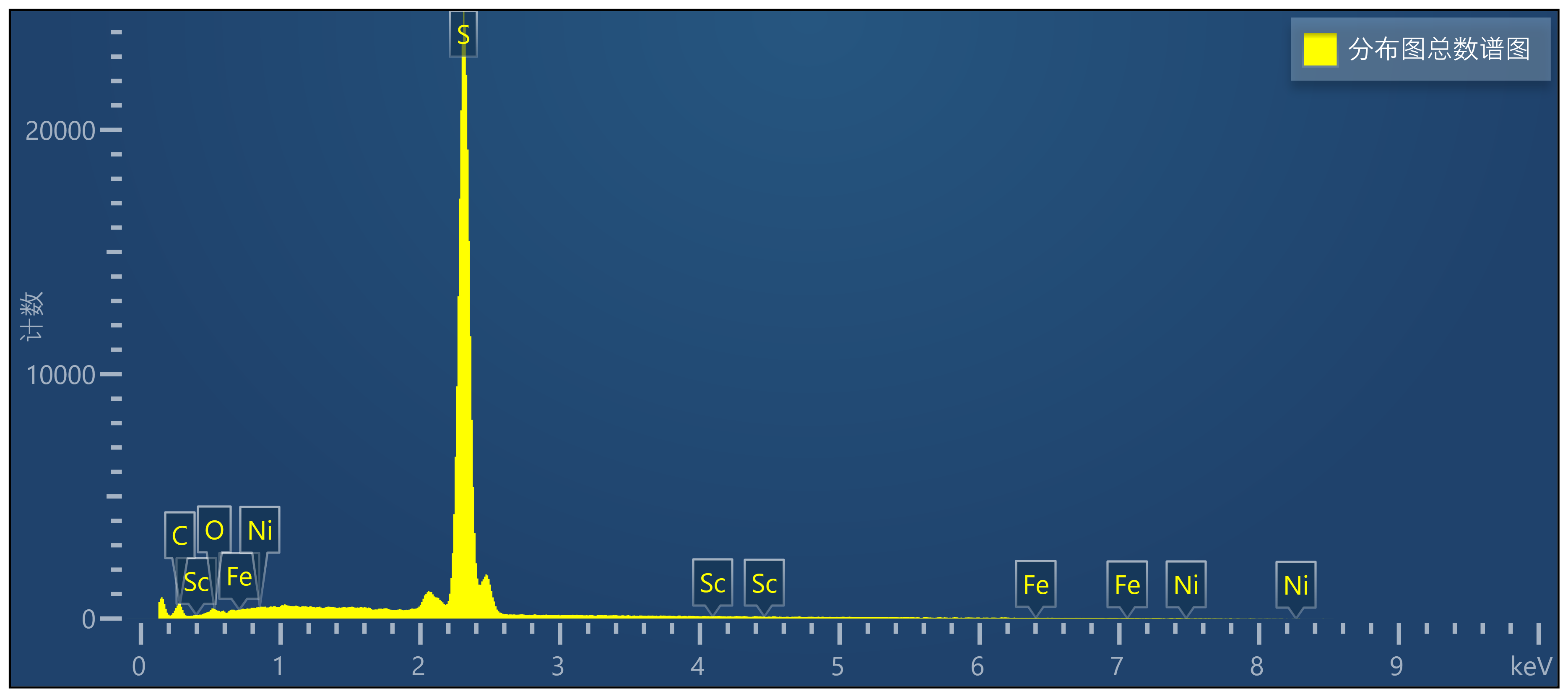


**Figure S32.** X-ray spectroscopy (EDX) analysis after the CE stability test.

| Element | Family | wt% | wt% Sigma | Atomic percent |
| --- | --- | --- | --- | --- |
| C | K | 26.59 | 1.05 | 48.81 |
| O | K | 1.09 | 0.13 | 1.51 |
| S | K | 72.14 | 1.04 | 49.61 |
| Sc | K | 0.03 | 0.08 | 0.02 |
| Fe | K | 0.00 | 0.40 | 0.00 |
| Ni | K | 0.15 | 0.14 | 0.06 |
| Total |  | 100 |  | 100 |


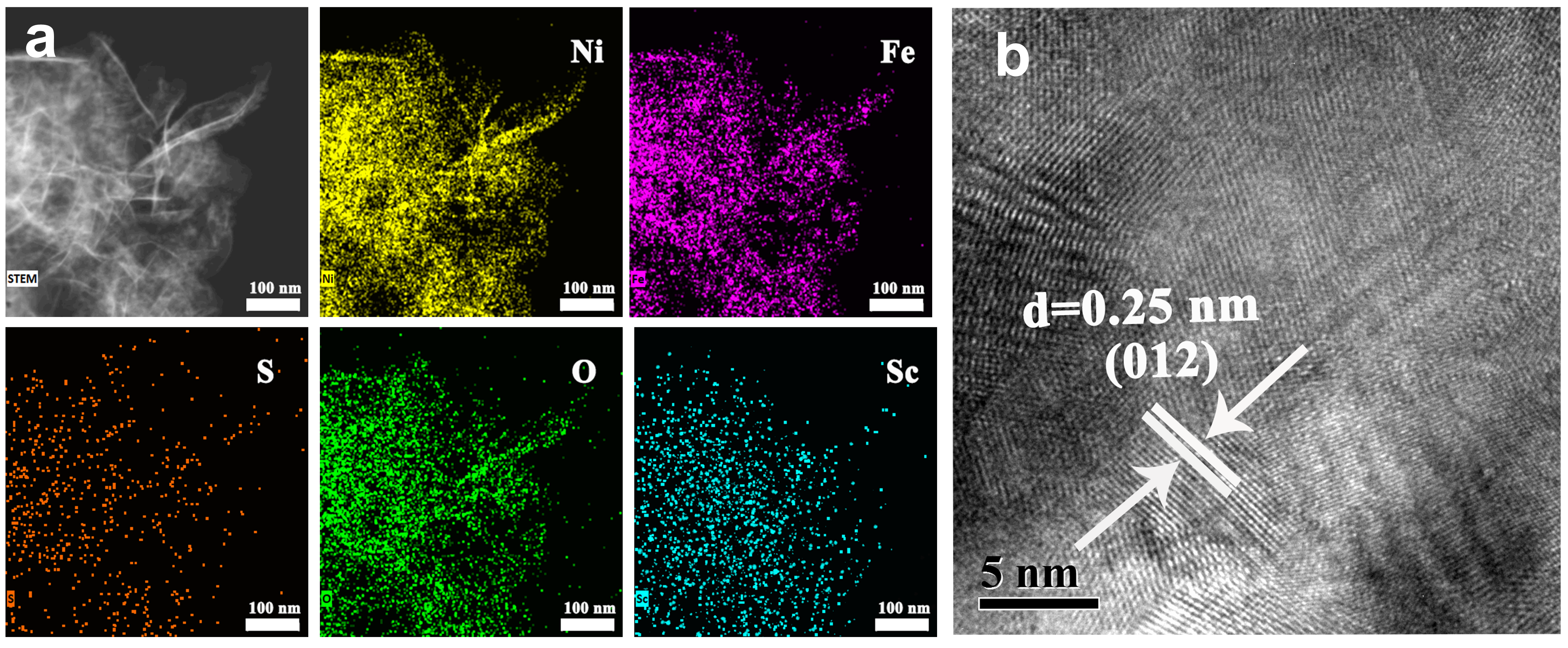


**Figure S33.** (a) Elemental mapping; (b) HRTEM after the PE stability test.


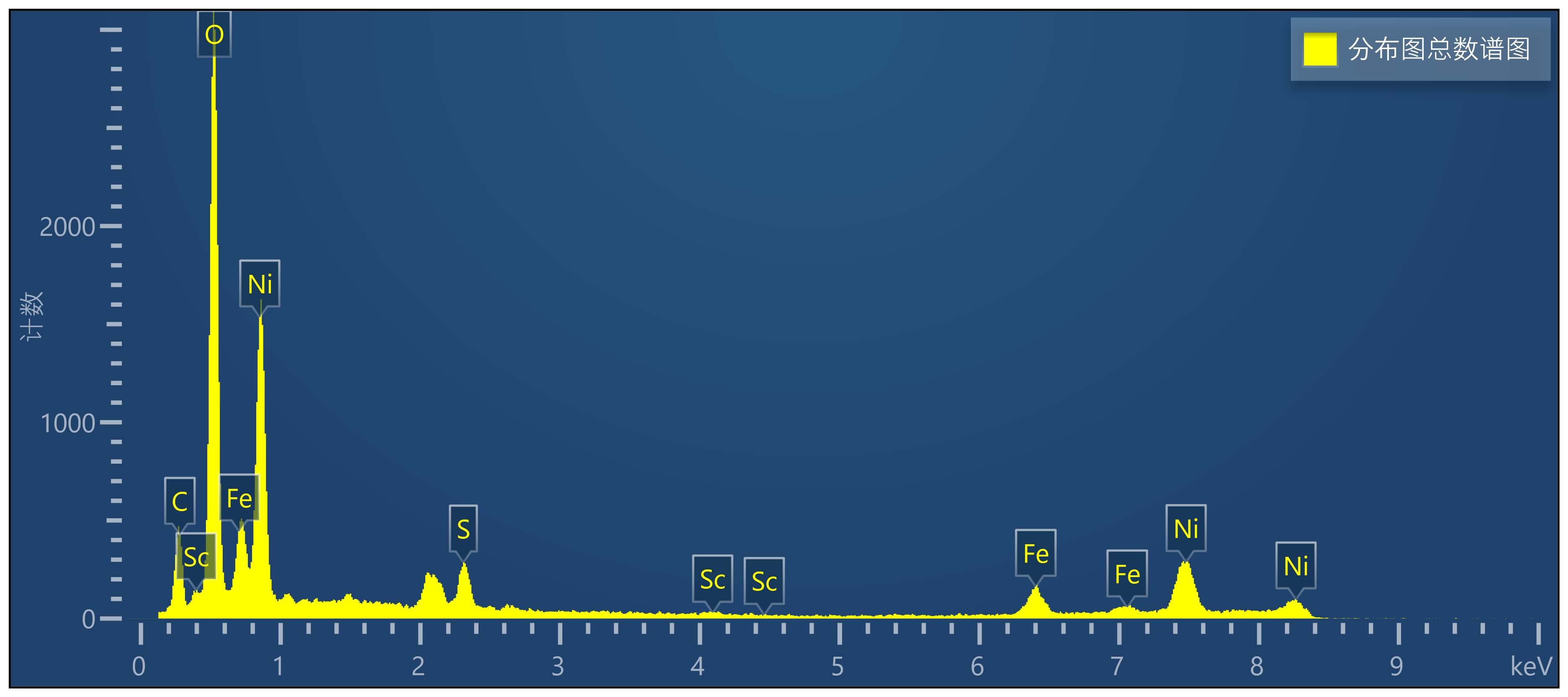


**Figure S34.** X-ray spectroscopy (EDX) analysis after the PE stability test.

| Element | Family | wt% | wt% Sigma | Atomic percent |
| --- | --- | --- | --- | --- |
| C | K | 16.42 | 0.75 | 32.49 |
| O | K | 30.16 | 0.55 | 44.79 |
| S | K | 1.99 | 0.11 | 1.62 |
| Sc | K | 0.51 | 0.12 | 0.16 |
| Fe | K | 15.76 | 1.00 | 6.71 |
| Ni | K | 35.16 | 0.69 | 14.23 |
| Total |  | 100 |  | 100 |

**Table S1.** EXAFS fitting parameters at the Fe&Ni *K*-edge and Sc L3-edge for various samples.

| Sample | Shell | CN^a^ | | R(Å)^b^ | σ^2^(Å^2^)^c^ | ΔE_0_(eV)^d^ | R factor | |
| --- | --- | --- | --- | --- | --- | --- | --- | --- |
| Fe *k-edge* | | | | | | | | |
| Fe foil | Fe-Fe | 8* | | 2.46±0.01 | 0.0052±0.0008 | 5.4±0.5 | 0.0055 | |
|  | Fe-Fe | 6* | | 2.84±0.01 | 0.0054±0.0009 | 4.6±0.7 |  |  |
| Fe_2_O_3_ | Fe-O | 6.0±0.6 | | 1.98±0.01 | 0.0133±0.0018 | -2.3±0.5 | 0.0026 | |
|  | Fe-Fe | 5.9±0.7 | | 2.98±0.01 | 0.0078±0.0010 | 3.2±0.4 |  |  |
|  | Fe-Fe | 2.3±0.6 | | 3.65±0.01 | 0.0007±0.0016 | -9.5±0.7 |  |  |
| NiFe-LDH | Fe-O | 6.0±0.1 | | 1.11±0.01 | 0.0038±0.0017 | 2.81±0.8 | 0.0042 | |
|  | Fe-M | 8.1±1.2 | | 3.11±0.01 | 0.0056±0.0022 | 1.71±0.5 |  |  |
| Sc-NiFe-LDH | Fe-O | 6.0±0.3 | 1.99±0.01 | | 0.0061±0.0008 | -2.8±0.3 | 0.0055 |  |
|  | Fe-M | 8.4±1.3 | 3.15±0.01 | | 0.0109±0.0013 | 5.1±0.5 |  |  |
|  | Fe-M | 4.1±1.2 | 3.47±0.01 | |  |  |  |  |
| Ni *K*-edge | | | | | | | | |
| Ni foil | Ni-Ni | 12* | | 2.48±0.01 | 0.0062±0.0003 | 6.9±0.2 | 0.0017 | |
| NiO | Ni-O | 6.0±0.7 | | 2.09±0.01 | 0.0072±0.0017 | -1.5±0.6 | 0.0053 | |
|  | Ni-Ni | 12.0±0.9 | | 2.95±0.01 | 0.0068±0.0006 | -3.8±0.3 |  |  |
| Sc-NiFe-LDH | Ni-O | 7.4±0.4 | | 2.04±0.01 | 0.0077±0.0009 | -3.4±0.3 | 0.0028 | |
|  | Ni-M | 9.3±0.9 | | 3.10±0.01 | 0.0102±0.0009 | 0.4±0.3 |  |  |
| Sc *L3-edge* | | | | | | | | |
| Sc foil | Sc-Sc | 12.0* | | 3.25±0.01 | 0.0078 | 3.9 | 0.0148 | |
| Sc2O3 | Sc-O | 6.0* | | 2.13±0.01 | 0.0059 | -2.4 | 0.0088 | |
|  | Sc-Sc | 6.0* | | 3.26±0.01 | 0.0038 | -8.4 |  |  |
| Sc-NiFe-LDH | Sc-O | 5.1±1.0 | | 2.15±0.01 | 0.0070 | 3.7 | 0.0158 | |

*^a^CN*, coordination number; *^b^R*, the distance to the neighboring atom; *^c^σ*^2^, Debye-Waller factor , the Mean Square Relative Displacement (MSRD); *^d^ΔE*_0_, inner potential correction; *R* factor indicates the goodness of the fit. *S*_0_^2^ was fixed to 0.750 and 0.783 respectively, accourding to the experimental EXAFS fit of Fe foil and Ni foil by fixing *CN* as the known crystallographic value. * This value was fixed respectively during EXAFS fitting, based on the known structure of Fe and Ni. Error bounds that characterize the structural parameters obtained by EXAFS spectroscopy were estimated as CN ± 20%; R ± 1%; σ2 ± 20%; ΔE0 ± 20%. A reasonable range of EXAFS fitting parameters: 0.700 < *Ѕ*_0_^2^ < 1.000; *CN >* 0; *σ*^2^ > 0 Å^2^; |Δ*E*_0_| < 15 eV; *R* factor < 0.02.

**Table S2.** Comparison of SOR performances of Sc-NiFe-LDH with other anode oxidation reactions

| **Catalysts** | **Electrolyte** | **Potential at 100 mA cm^-2^ (V)** | **Power consumption (kWh m^-3^ H_2_)** | **Max flow rate of H_2_ production (mL min^-1^)** | **Tafel plots**  **(mV dec^-1^)** | **Operation stability (h)** | **Reference** |
| --- | --- | --- | --- | --- | --- | --- | --- |
| Sc-NiFe-LDH | 1.0 m NaOH + 1.0 m Na_2_S | 0.9 | 2.19 | 5.43 | 13.5 | 530 | This work |
| WS_2_ NSs/CP | 1.0 m NaOH + 2.0 m Na_2_S | 1.32 | 3.16 | 0.6 | / | 192 | *Angew. Chem. Int. Ed.* **2021,** 60, 21550-21557. |
| NiSe | 1.0 m NaOH + 1.0 m Na_2_S | 0.49 | 2.63 | 4.62 | / | 500 | *Applied Catalysis B: Environmenta,* ***2023****, 324,122255* |
| CoS2@C/MXene/NF | 1.0 m NaOH + 1.0 m Na_2_S | 0.6 | 2.32 | 0.49 | 64.5 | 240 | Adv. Mater. **2022**, 34, 2109321. |
| Cu_2_S/NF | 1.0 m NaOH + 1.0 m Na_2_S | 0.64 | / | 0.37 | 68 | 48 | Green Chem., **2021**, 23, 6975 |

| **Table S3.** Comparison of the *Sc-NiFe-LDH* assembled electrolyzer system with previously reported overall water splitting systems in cell performance. | | | | | | |
| --- | --- | --- | --- | --- | --- | --- |
| **Catalysts** | **Electrolyte** | **Cell voltage at 100 mA cm^-2^ (V)** | **Power consumption (kWh m^-3^ H_2_)** | **Reaction** | **Operation stability (h)** | **Reference** |
| Sc-NiFe-LDH | 1.0 m NaOH + 1.0 m Na_2_S | 0.9 | 2.19 | HER/SOR | 530 | This work |
| WS_2_ NSs/CP | 1.0 m NaOH + 2.0 m Na_2_S | 1.32 | 3.16 | HER/SOR | 192 | *Angew. Chem. Int. Ed.* **2021**, 60, 21550-21557 |
| Ni_2_P-Fe_2_P | 1.0 m KOH seawater | 1.80 | 4.31 | HER/OER | 48 | *Adv. Funct. Mater.* **2020***,* 31, 2006484 |
| CoPx@FeOOH | 1.0 m KOH +  0.5 m NaCl | 1.688 | 4.034 | HER/OER | 80 | Appl. Catal.  B **2021**, 294, 120256. |
| Mo-NiPx/NiSy | 1.0 m KOH | 1.7 | 4.063 | HER/OER | 27 | Adv. Funct. Mater. **2021**, 31, 2101532 |
| h-NiMoFe | 1.0 m KOH | 1.43 | 3.418 | HER/OER | 40 | Energy Environ. Sci. **2021**, 14, 4610-4619 |
| MoO_2_-FeP@C | 1.0 m KOH + 1.0 m HMF | 1.70 | 4.07 | HER/HMFOR | / | *Adv. Mater.* **2020,** 32, e2000455 () |
| CoP film | 1.0 m KOH +50 mm HMF | / | 3.75 | HER/HMFOR | / | *ACS Energy Lett.* ***2016****, 1, 386−390* |
| *n*-Co_3_S_4_@NF | 1.0 m NaOH seawater | 1.57 | 3.77 | HER/OER | 504 | *Nat. Commun.* ***2024****,15:6173.* |
| NiMoO-Ar | 1 m KOH+ 0.5 m urea | 1.58 | 3.75 | HER/UOR | 50 | *Energy Environ. Sci.,* ***2018****, 11, 1890-1897* |
| CoNi@CN‐CoNiMoO | 1.0 m KOH+ 0.5 m urea | 1.39 | 3.34 | HER/UOR | 120 | *Carbon Ener.*, **2023**, e368 |
| NiFeOx-NF | 1.0 m KOH + 0.5 m glucose | 1.39 | 3.33 | HER/GOR | 24 | *Nat. Commun.* **2020,** 11, 265 () |
| Fe_0.1_-CoSe_2_/CC | 1.0 m KOH + 0.5 m glucose | 1.68 | 4.02 | HER/GOR | 20 | *Appl. Catal., B* **2020,** 277, 119178 |
| NC@CuCo_2_Nx/CF | 1.0 m KOH + 0.5 m benzyl alcohol | 1.82 | 4.37 | HER/BOR | 60 | *Adv. Funct. Mater.* **2017**, 27, 1704169 |
| Co-Se1 | 1.0 m KOH | 1.51 | 4.31 | HER/OER | 12 | *Adv. Energy Mater.* ***2018****, 8, 1801926* |
| S-(Ni, Fe)OOH | 1.0 m KOH+Seawater | 1.661 | 3.9 | HER/OER | 100 | *Energy Environ. Sci.* ***2020****, 13, 3439* |
| NiFe-LDH | 1.0 m KOH+Seawater | / | 3.83 | HER/OER | 100 | *Adv. Energy Mater.* ***2018****, 8, 1800338* |
| NiNS | 1.0 m KOH+Seawater | 1.95 | 4.31 | HER/OER | 12 | *J. Mater. Chem. A,****2019****,7, 8117–8121* |
| NiFe/NiS*x*/NF | 6.0 m NaOH +1.5 M NaCl 80 ℃ | 1.5 | 4.11 | HER/OER | 1000 | *PNAS* ***2019,*** *116, 6624* |
| Fe*x*&Mo-NiO | 6 m KOH + seawater 60 °C | 1.545 | 3.59 | HER/OER | 80 | *Energy Environ. Sci.* ***2022****, 15, 3945* |
| SSM | 1.0 m KOH +seawater | 1.635 | 4.27 | HER/OER | 200 | *Adv. Mater.* ***2022****, 34 2201774* |

**Table S4.** Calculation details of energy equivalent input and CO_2_ equivalent emission for hybrid seawater-SOR electrolyzer system and alkaline water electrolysis.

| **Content** | **Alkaline water electrolysis** | | **Hybrid seawater-SOR electrolyzer system** | |
| --- | --- | --- | --- | --- |
|  | **CO_2_ equivalent emission**  **(t_CO2_ t_H2_^−1^)** | **Energy equivalent input (GJ t_H2_^−1^)** | **CO_2_ equivalent emission**  **(t_CO2_ t_H2_^−1^)** | **Energy equivalent input (GJ t_H2_^−1^)** |
| Water extraction & deionization | 0.045 | 0.000117 | 0.045 | 0.000117 |
| Heating H_2_O to 70°C | 0.01 | 1.696 | 0 | 0 |
| Electrolysis stack | 1.04 | 187.2 | 0.52 | 93.8 |
| Heat demand of reaction | 0.151 | 27.25 | 0 | 0 |
| Overall | 1.246 | 216.146 | 0.565 | 93.80 |

Note. The data for alkaline water electrolysis, electrochemical methane splitting, and natural gas steam reforming are obtained from *Nature Communications (2024, 15, 6173)*, the data for the hybrid seawater-SOR electrolyzer system are obtained from *Advanced Materials (2022, 34, 2109321)*.

**Table S5**. Elemental analysis (EA) results for C, H, N, and S.

| No. | N (%) | C (%) | H (%) | S (%) |
| --- | --- | --- | --- | --- |
| 1 | 0.00 | 0.00 | 0.21 | 99.53 |
| 2 | 0.00 | 0.00 | 0.23 | 99.56 |

**3.** **Calculation of techno-economic analysis**

A specific sample was selected for the TEA calculation of hydrogen and sulfur production.

Taking the 100 tones daily capacity of H_2_ as an example at a current density of 100 mA cm^-2^ with 100% faradaic efficiency of H_2_ as an example.

The specific assumptions made for the TEA are listed as follows:

1. The electrolyser cost is assumed to be $10,000 per m^2^, with a plant lifetime of 20 years and an annual operation time of 350 days.
2. The total cell operating voltage is 1.7 V.
3. The price of electricity is considered to be 0.03 $ kWh^-1^.
4. The total catalyst and membrane cost are assumed to be 5% of electrolyzer cost.
5. The cost of separation is set at 10% of the electricity costs.
6. The maintenance cost is assumed 5% of the capital costs.
7. The balance of plant (BOP) capital cost is assumed 50% of the capital costs.
8. The cost of operating supplies for the process is set at 10% of the capital costs.

**Table S6** Values of feedstocks and products.

| Product | Values ^a, b^（$ ton^-1^） |
| --- | --- |
| Feedstocks | |
| NaOH | 240 |
| H_2_O | 0.22 ^[4]^ |
| Formic acid | 400 ^[4]^ |
| Products | |
| S | 220 ^[5]^ |
| HCOONa | 4750 |
| H_2_ | 1900 ^[6]^ |

a Value data are obtained and calculated by ref^4-6^ and the website of https://jiage.molbase.cn.

b The origin prices of chemicals on the website were dominated in RMB, and the US dollar calculated here was based on a unit of exchange rate of 7.2 (average on 2023 and 2024; <https://gushitong.baidu.com/foreign/global-USDCNY>)

Thus, the parameters needed in the cost calculation are determined as follows：

Assuming a production rate of 100000 kg day^-1^, the current needed is:

$$100000\frac{kg}{day}\times\frac{day}{86400s}\times1000\frac{g}{kg}\times\frac{mol}{18}\times{2e}^{-}\times96485\frac{C}{mol}=12408050 A$$

The electrolyzer area needed is the total current divided by the current density. According to the current required and the assumed operating current density of 100 mA cm^-2^, we can calculate the area of electrolyser needed:

$$Electrolyzer area=\frac{12408050}{0.1 \frac{A}{{cm}^{2}}}\times\frac{m^{2}}{{10}^{4} {cm}^{2}}=12408.05 m^{2}$$

The power needed is given from P=VI: (Here we take the 1.48 V as the potential)

$$Power=1.7 V\times12408050 A\times\frac{W}{{10}^{6} MW}=21.09 MW$$

1. **Capital Costs**

The electrolyser cost can be calculated based on the estimate of $10000 per m^2^,

1. The electrolyzer capital cost per ton of sulfur:

$$Electrolyzer cost=12408.05 m^{2} \times\frac{\$10000}{m^{2}}\times\frac{1}{20 year}\times\frac{year}{350 day}\times\frac{day}{100 ton}=177.25 \$ {ton}^{-1}$$

1. Catalyst and membrane:

The total catalyst and membrane cost are assumed to be 5% of electrolyzer cost and is calculated as: Cost of catalyst and membrane =$177.25\frac{\$}{\mathrm{ton}}\times5\%=8.86 \${ton}^{-1}$

1. Hydrolyzer and distillation equipment are calculated as:

According to the process flow diagram, capital cost of equipment is considered as:^[7]^

Capital cost of equipment= Flash Tank + Neutralization Reactor + MSMPR Crystallizer + Rotary Vacuum filter=$\$$142500+$\$$251500*4+$\$$970400+$\$$256968=$\$$3146772

𝑆𝑒𝑝𝑎𝑟𝑎𝑡𝑖𝑜𝑛 𝐸𝑞𝑢𝑖𝑝𝑚𝑒𝑛𝑡 𝐶𝑜𝑠𝑡 $=\$3146772 \times\frac{\$10000}{m^{2}}\times\frac{1}{20 year}\times\frac{year}{350 day}\times\frac{day}{100 ton}$=4.49 $${ton}^{-1}$

1. Separation:

The cost of separation is set at 10% of the electricity costs and is calculated as: Separation=151.87 × 10%=15.18 $${ton}^{-1}$

$Total capital cost=Electrolyzer capital cost+Catalyst and membrane+Separation Equipment+\mathrm{Separation}=177.25+8.86+4.49+15.18=205.80$ $${ton}^{-1}$

1. **Operating Cost**
2. Electricity:

The electricity cost per ton of sulfur is calculated based on the required power consumption and the unit price of electricity:

$$Electricity cost=21.09 MW\times\frac{{10}^{3} MW}{kW}\times24 h\times\frac{\$0.03}{kWh}\times\frac{1 day}{100 ton}=151.87 \${ton}^{-1}$$

2) Maintenance:

The maintenance cost is assumed 5% of the capital costs and is calculated as:

Maintenance cost per day = 205.80× 5%=10.29 $

3) Balance of Plant:

The balance of plant (BOP) capital cost is assumed 50% of the capital costs and is calculated as: Balance of Plant = 205.80× 50%=102.9 $

4) Operating supplies:

The cost of operating supplies for the process is set at 10% of the capital costs and is calculated as: Operating supplies = 205.80× 10%=20.58 $

$$Total operating cost=Electricity+Maintenance+Balance of Plant+Operating supplies=151.87+10.29+102.9+20.58=285.64$$

1. **Chemical reagents Cost**

Cost of chemical reagents= NaOH cost + Water cost+ FA cost =$8.95+$0.205+$55.95=$65.11

$$Cost of chemical reagents=\frac{\left( 37300\times\$0.24+932600\times\$0.00022+139980\times\$0.4 \right)}{1000}=65.11 \${ton}^{-1}$$

𝑇𝑜𝑡𝑎𝑙 𝑐𝑜𝑠𝑡 =205.80+285.64+65.11=556.56 $\${ton}^{-1}$

The products of this process can obtain S, H_2_, and sodium formate. Therefore, the product value can be calculated as:

$$Product income=\frac{\left( 100000\times\$1.9+6332\times\$0.22+156501\times\$4.75 \right)\times0.95}{1000}=896.58 \${ton}^{-1}$$

In the production, due to the limited lifetime of the catalyst, continuous replacement of the catalyst must be considered to maintain sustained operation. Therefore, the catalyst lifetime will significantly affect the overall capital cost of the electrolyzer. Given a catalyst lifetime of 500 hours (i.e., 20 days), a production scale of 100 tons of hydrogen per day, an electrolyzer lifetime of 20 years, an annual operating time of 350 days, a catalyst cost of 50 $ m^-2^, and a total electrolyzer area of 12,408.05 m^2^, the calculation of the catalyst cost proportion is as follows:

Total electrolyzer cost (excluding catalyst):

Cost of stack=10000 $ m^-2^ ×12408.05 m^2^=124080500 $

Number of catalyst replacements:

$N=\frac{20\times350}{20}$**=**350

Total catalyst cost=350 × 50 $ m^-2^×12408.05 m^2^=217141000 $

Catalyst cost proportion=$\frac{217141000}{124080500+217141000}$×100%=63.5%


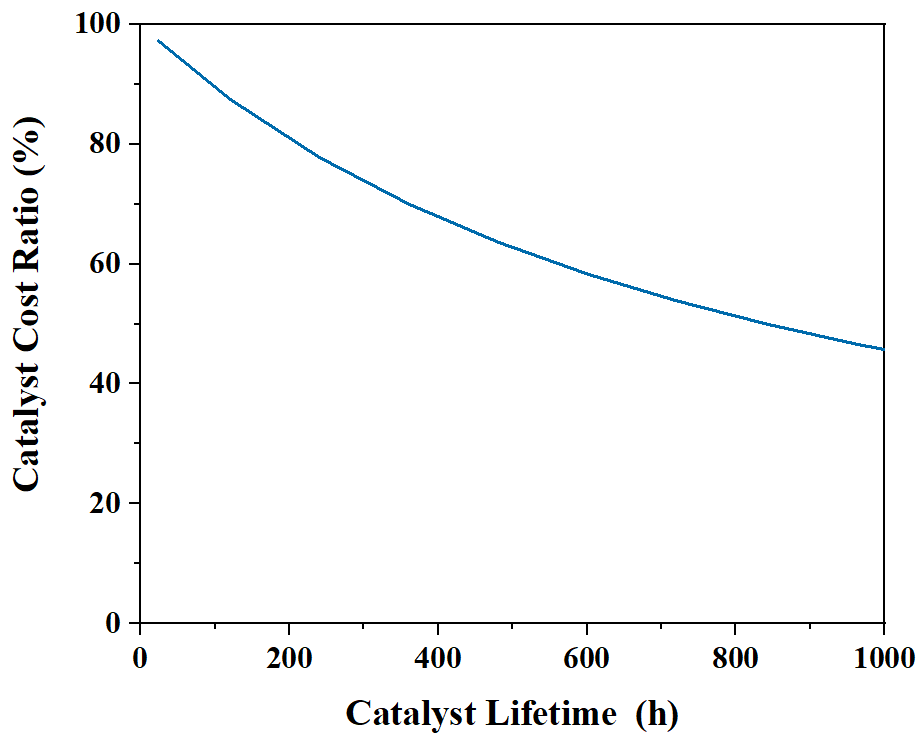


**Figure S35.** Relationship between catalyst lifetime and its proportion of the total electrolyzer cost.

**4. Outlook:**

The pulsing strategy demonstrated here possesses broad applicability beyond the sulfur oxidation system. By dynamically modulating the catalyst’s coordination environment, it effectively prevents active-site passivation, promotes continuous site regeneration, and accelerates reaction kinetics—mechanistic advantages that are not confined to a specific catalyst or reaction type. In principle, this approach can be extended to a wide range of electrocatalytic reactions involving surface-bound intermediates or strongly adsorbed species, such as electrocatalytic nitrate reduction, alcohol oxidation, and CO_2_ electroreduction. More generally, the concept exemplifies how potential-driven dynamic control can fundamentally reshape catalytic pathways, providing a universal route for constructing self-regenerative and high-performance electrocatalysts across diverse applications.


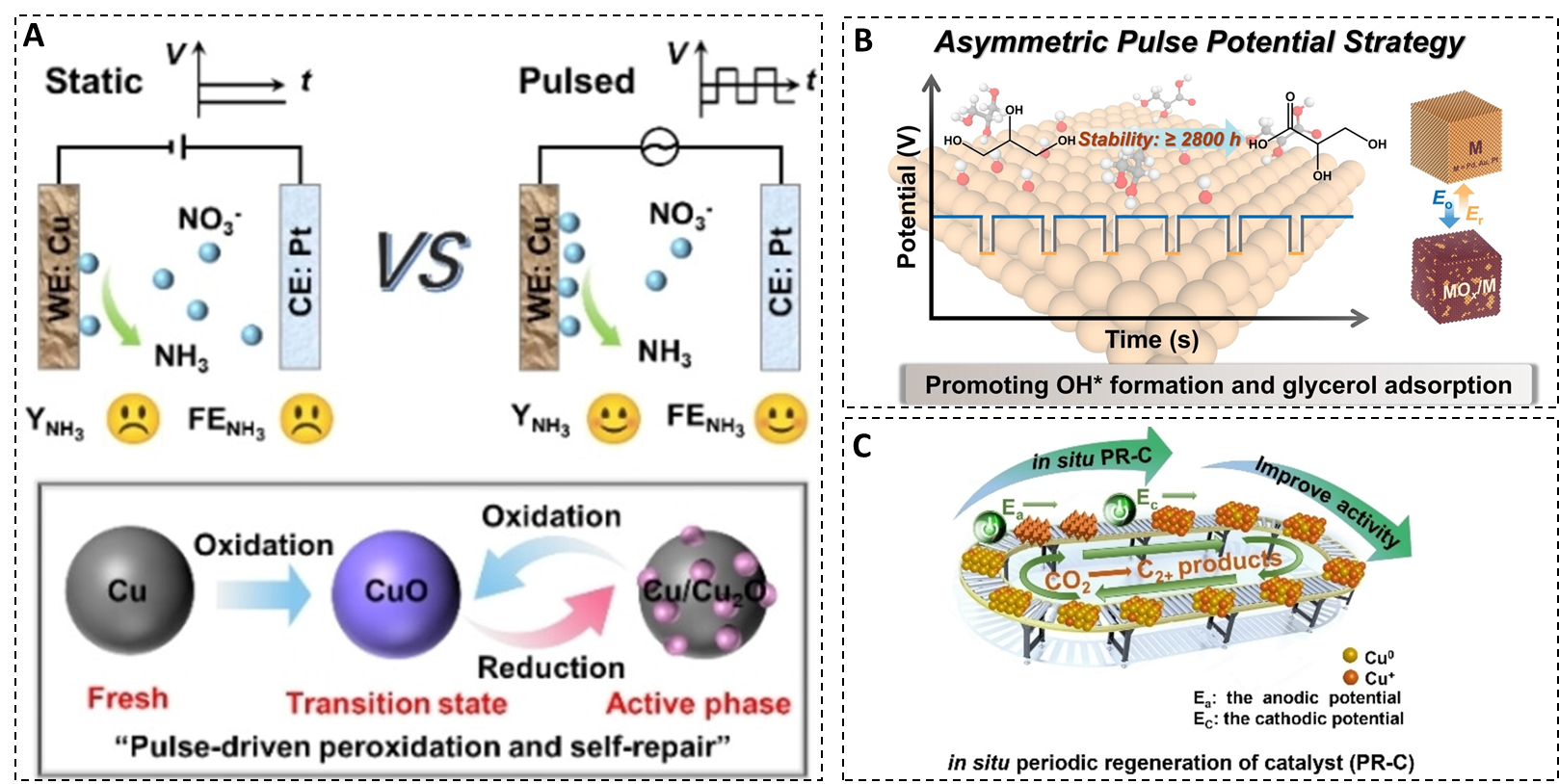


**Figure S36.** (A) Electrocatalytic nitrate reduction sustainably produces ammonia; (B) Promoting alcohols electrooxidation coupled with hydrogen production via asymmetric pulse potential strategy (C) In situ periodic regeneration of catalyst during CO_2_ electroreduction to C_2+_ Products.

The generalized applicability of this pulsing strategy has been demonstrated in multiple systems.
**(A)** In *electrocatalytic nitrate reduction*, a programmable pulsed potential maintains a dynamic Cu/Cu₂O structure, periodically oxidizing Cu to CuO and subsequently regenerating Cu/Cu₂O during reduction. This modulation breaks the rate-determining NO_3_^−^→NO_2_⁻ step and suppresses the competing hydrogen evolution reaction, yielding superior NH₃ production performance.
**(B)** In *alcohol oxidation*, an asymmetric pulse potential promotes the continuous regeneration of active oxygen species, thereby stabilizing noble-metal catalysts (e.g., Pd) and extending stable glycerol oxidation operation from 6 h to over 2800 h.
**(C)** In *CO₂ electroreduction*, a periodic regeneration strategy (PR-C) applies short positive potential pulses in halide-containing electrolytes, continuously restoring the Cu surface oxidation state and structure to maintain high selectivity for C_2+_ products.

Together, these examples highlight that the pulsing process is not merely a system-specific optimization but a general mechanistic paradigm for dynamic catalyst regulation—transforming static surface reactions into self-renewing catalytic cycles with broad implications for sustainable electrochemical synthesis.

**References**

[1] S. Zhang, Q. Zhou, Z. Shen, X. Jin, Y. Zhang, M. Shi, J. Zhou, J. Liu, Z. Lu, Y.-N. Zhou, H. Zhang, “Sulfophobic and vacancy design enables self-cleaning electrodes for efficient desulfurization and concurrent hydrogen evolution with low energy consumption” *Adv. Funct. Mater.* **2021**, *31*, 2101922.

[2] Y. Pei, D. Li, C. Qiu, L. Yan, Z. Li, Z. Yu, W. Fang, Y. Lu, B. Zhang, “High-entropy sulfide catalyst boosts energy-saving electrochemical sulfion upgrading to thiosulfate coupled with hydrogen production” *Angew. Chem., Int. Ed.* **2024**, *63*, e202411977.

[3] Y. Wu, Z. Hou, C. Wang, “Construction of an Sc-NiFe-LDH electrocatalyst for highly efficient electrooxidation of 5-hydroxymethylfurfural at industrial current density” *Nanoscale* **2025**, *17*, 3114–3122.

[4] M. Song, Y. Wu, Z. Zhao, M. Zheng, C. Wang, J. Lu, “Corrosion Engineering of Part-Per-Million Single Atom Pt1/Ni(OH)_2_ Electrocatalyst for PET Upcycling at Ampere-Level Current Density” *Advanced Materials* **2024**, *36*, 2403234.

[5] X. Teng, K. Shi, L. Chen, J. Shi, “Coupling electrochemical sulfion oxidation with CO_2_ reduction over highly dispersed p‐Bi nanosheets and CO_2_‐assisted sulfur extraction” *Angew. Chem., Int. Ed.* **2024**, *136*, e202318585.

[6] W. R. Leow, Y. Lum, A. Ozden, Y. Wang, D.-H. Nam, B. Chen, J. Wicks, T.-T. Zhuang, F. Li, D. Sinton, E. H. Sargent, “Chloride-mediated selective electrosynthesis of ethylene and propylene oxides at high current density” *Science* **2020**, *368*, 1228–1233.

[7] M. C. Massaro, A. H. A. Monteverde, “Techno-economic analysis of FDCA production through electrocatalytic processes” *J. Electrochem. Soc.* **2022**, *169*, 054515.
